# Proteomic Analysis of Decellularized Extracellular Matrix: Achieving a Competent Biomaterial for Osteogenesis

**DOI:** 10.1155/2022/6884370

**Published:** 2022-10-11

**Authors:** Gabriela M. Monteiro-Lobato, Pedro S. T. Russo, Flavia V. Winck, Luiz H. Catalani

**Affiliations:** ^1^Departamento de Química Fundamental, Instituto de Química, Universidade de São Paulo, São Paulo, Brazil; ^2^Departamento de Análises Clínicas e Toxicológicas, Faculdade de Ciências Farmacêuticas, Universidade de São Paulo, São Paulo, Brazil; ^3^Departamento de Bioquímica, Instituto de Química, Universidade de São Paulo, São Paulo, Brazil

## Abstract

Decellularized ECMs have been used as biological scaffolds for tissue repair due to their tissue-specific biochemical and mechanical composition, poorly simulated by other materials. It is used as patches and powders, and it could be further processed via enzymatic digestion under acidic conditions using pepsin. However, part of the bioactivity is lost during the digestion process due to protein denaturation. Here, stepwise digestion was developed to prepare a competent biomaterial for osteogenesis from three different ECM sources. In addition, three different proteases were compared to evaluate the most effective digestion protocol for specific cellular processes. GAGs and peptide quantification showed that the stepwise method yielded a higher concentration of bioactive residues. Circular dichroism analysis also showed that the stepwise approach preserved the secondary structures better. The protein profiles of the digested ECMs were analyzed, and it was found to be highly diverse and tissue-specific. The digestion of ECM from pericardium produced peptides originated from 94 different proteins, followed by 48 proteins in ECM from tendon and 35 proteins in ECM from bone. In addition, digested products from pericardium ECM yielded increased proliferation and differentiation of bone marrow mesenchymal stem cells to mature osteoblasts.

## 1. Introduction

The extracellular matrix (ECM) is a noncellular biological material present between tissues and organs with a complex 3D structure, composed mainly of a network of proteins and polysaccharides that are continually being produced, secreted, and reshaped in dynamic equilibrium [1–3]. Thus, the ECM continually changes according to cellular needs and environmental conditions. In other words, the ECM is more than mechanical support, having an active role in cell regulation and survival [2, 4].

Over the past decade, decellularized ECMs have been used as biological scaffolds for tissue repair due to their tissue-specific biochemical and mechanical composition, which is poorly simulated by other biomaterials [3, 5]. Historically, decellularized ECMs have been used as patches for surgical procedures and powders for injection and catheter-based procedures [3].

The decellularization process employs a series of agents and protocols to avoid or minimize any adverse immunologic response. The ultimate goal is to remove all cellular material and residues without affecting the biochemical composition, mechanical structure, and possible bioactivity. However, studies have shown that several adversities are encountered throughout the decellularization process, such as collagen degradation and GAG removal, resulting in loss of mechanical and viscoelastic properties, respectively [1]. Moreover, decellularized ECMs could be further processed via enzymatic digestion under acidic conditions using pepsin and reassembled into an injectable hydrogel once brought to physiological conditions [6]. During this long digestion process, it is likely that the degradation of bioactive molecules occurs, like proteins, peptides, and growth factors responsible for recruiting stem cells, triggering their differentiation, and promoting tissue formation and remodeling. According to Williams, cardiomyocyte proliferation increases when cultured with cardiac ECM digested for 1 h to 3 h, compared with the same ECM digested for more extended periods. Also, the proteomic analysis of each set of partially digested ECM showed a different protein profile: 3 h digestion was mainly composed of fibrillin-1, fibrinogen, collagen I, and laminins, while longer digestions increased the amount of collagen I [7]. That led us to hypothesize that prolonged (up to 24 h to 48 h) digestion to which the decellularized ECM is subjected to allow processing is a major operation responsible for protein denaturation, especially of some important cell signaling proteins.

Therefore, owing to the excellent biological responses achieved during *in vitro* and *in vivo* use of decellularized ECM in medicine [6–17], we envisage the idea of carefully dismantling its 3D structure, avoiding continuous digestions, hoping to preserve bioactive molecules. The product thus formed is a promising material in tissue engineering and cell regeneration applications, especially when coupled with other techniques such as electrospinning [18], coating of materials [19–21], mixing gels with cells [22–24], and development of bioinks for 3D printing [25–27].

The great advantage of this strategy is that for most of the aforementioned techniques, the gel assembly is not necessary, and therefore, the standard 24 h ECM digestion is not required, enabling new digestion methods to be evaluated to preserve the maximum bioactivity of the new biomaterial. Additionally, 3D printing techniques enable the fabrication of tailor-made complex structures with high precision in micron and submicron scales [27, 28].

Here, we developed a new discontinued protocol (stepwise digestion), which renders a more integral mixture of peptides, maintaining a significant part of some molecules' active sites and binding ligands. In addition, we compare the biological effect of standard (continuous) and stepwise digestion products—from three different proteases—when incubated with mesenchymal stem cells, aiming at materials more suited for specific cell responses like cell adhesion, proliferation, and osteogenic differentiation. Thus, this work presents and compares a new possibility to customize the digestion processes to achieve the optimal biochemical composition for tissue engineering materials, especially for studies regarding bone healing.

## 2. Materials and Methods

### 2.1. ECM Digestion

Bovine ECMs derived from the demineralized cortical bone (ECMb, Baumer), pericardium (ECMp, HPBio), and tendon (ECMt, Sigma-Aldrich) were digested with three different protease solutions, as described below:

- Collagenase from *Clostridium histolyticum* Type IA-S (Sigma-Aldrich) in TRIS-HCl buffer (10 mM) containing CaCl_2_ (2 mM), pH 7.4 buffer [29]
- Pepsin from porcine gastric mucosa (Sigma-Aldrich) in 0.1 M HCl solution [13]
- Trypsin from bovine pancreas (Sigma-Aldrich) in PBS pH 7.4 [30]

ECMt and ECMb were not pretreated before digestion. However, due to its commercial condition, ECMp was washed three times, freeze-dried, and milled in a ball-mill Pulverisette 6 (Fritsch) for 5 min at 600 rpm before digestion.

Each ECM sample was digested eighteen times, changing one of the significant variables analyzed, as (a) ECM origin (ECMb, ECMt, and ECMp), (b) protease type (collagenase, trypsin, and pepsin), and (c) digestion method (continuous and stepwise).

#### 2.1.1. Continuous Digestion

ECM (10 mg/mL) was added to one of the specific enzymatic solutions (1 mg/mL) [7]. The digestion process was performed at room temperature in an orbital shaker at 100 rpm for 24 h.

#### 2.1.2. Stepwise Digestion

ECM (10 mg/mL) was added to one of the specific enzymatic solutions (1 mg/mL). The digestion process was performed at room temperature in an orbital shaker at 100 rpm at different time intervals. During 24 h of incubation, the digestion was stopped periodically (after 1 h, 3 h, 6 h, 8 h, and 24 h after starting the experiment), and the supernatant was collected and frozen. To the remaining solid material, an extra loading of the enzymatic solution was added to further continue the digestion, maintaining the initial concentration of ECM (10 mg/mL).

### 2.2. Quantification and Structure Identification of Proteins and GAGs

According to the manufacturer's instructions, the concentration of peptides yielded after digestion was quantified using the MicroBCA assay (ThermoFisher, USA). Absorbance was quantified on a colorimetric plate reader at 540 nm (Infinite 200 Pro NanoQuant—Tecan, Switzerland), converted to protein concentration using standards provided by the kit.

Glycosaminoglycans (GAGs) in solution were quantified using dimethyldimethylene blue staining (DMMB), as previously described [31]. A digested ECM (20 *μ*L) sample was added to the DMMB solution (200 *μ*L). Absorbance was quantified on a colorimetric plate reader at 535 nm (Infinite 200 Pro NanoQuant—Tecan, Switzerland).

In order to evaluate the presence of protein secondary structures, samples of digested ECM were analyzed using circular dichroism (JW720, Jasco, Japan) between 190 nm ≤ *λ* ≤ 300 nm, 20 nm/min, and accumulation was set in 3.

### 2.3. ECM Proteomics

The proteomic analysis was conducted on the whole ECMs (before digestion) using samples of ECMp, ECMt, and ECMb. The commercial ECMs were solubilized with urea buffer (urea (6 M), thiourea (2 M), pH 8, and phenylmethylsulfonyl fluoride (PMSF)) sonicated and centrifuged (10 min at 10000 rpm at 4°C) to separate contaminants. The proteins contained in the supernatant were quantified using the Bradford method [32] followed by reduction (dithiothreitol (5 M) for 25 min at 56°C), alkylation (2-iodoacetamide (14 mM) for 30 min at room temperature in the dark), quenching of free iodoacetamide (DTT (5 mM) for 15 min at room temperature in the dark), 1 : 5 *v*/*v* dilution with ammonium bicarbonate (50 mM), and digestion with sequencing grade modified trypsin (Promega) (1 : 50 *w*/*w* for 16 h at 37°C). The reaction was stopped with 0.4% TFA. The samples were desalted using the Stage Tips method as previously described [33].

LC-MS/MS analyses were processed as follows: the peptides were diluted in formic acid (0.1%) and analyzed on a q-Tof Maxis 3G spectrometer (Bruker Daltonics, USA) coupled with a Nano LC Acquity instrument (Waters, USA). The ion source used in the mass spectrometer was the CaptiveSpray (Bruker Daltonics, USA). The electrospray voltage was set to 2 kV, and the source temperature was 150°C. Peptide separation was performed in two steps: trapping and analytics. The trapping step was performed using the nanoAcquity UPLC® 2G-V/MTrap Symmetry® C18 column (180 *μ*m × 20 mm, 5 *μ*m) for 3 min at a flow rate of 7 *μ*L/min with formic acid (0.1%). It was followed by the analytical step, using nanoAcquity UPLC® BEH130 (100 *μ*m × 100 mm, 1.7 *μ*m) and acetonitrile with formic acid (0.1%) as a mobile phase in a gradient of 2–85% in formic acid (0.1%) at a flow rate of 0.3 *μ*L/min for 265 min. The mass spectrometry raw data is deposited in the PeptideAtlas® repository and can be accessed by the code (PASS01440).

The protein identification was performed using the Andromeda® search algorithm within the MaxQuant® v.1.5.6.5 software platform against the UniProt® Protein Database for *Bos taurus* (release: September 25, 2017, 23,969 sequences). The search parameters were set to a maximum of two missing cleavages by the trypsin enzyme, with a maximum error tolerance of 40 ppm for MS search and 0.01 Da for MS/MS search. A decoy protein database with reverse peptide sequences was used to quantify and identify proteins keeping a maximum 1% FDR at peptide and protein levels. All proteins were identified by at least one unique peptide.

On a total proteome analysis, acetylation, oxidation, and hydroxyproline were set as variable modifications, and carbamidomethylation was set as fixed modification. A second analysis was performed for the identification and quantification of proteins using the same parameters as above, but including the following possible variable posttranslational modifications: Cys-Cys, deamidation (N), hydroxyproline, oxidation (M), phosphorylation (ST), and sulfation (Y) [34]. For both analyses, protein quantification was performed using a previously described label-free quantification (LFQ) algorithm implemented in the MaxQuant® software platform [35]. Data preprocessing analysis was performed using Perseus® v.1.3.0.4 software to exclude reverse sequence identification and only identified by site entries.

Functional annotation of the identified proteins was performed using Gene Ontology (GO) annotation from *Bos taurus* species. Enrichment analysis of the overrepresented GO terms for the list of proteins identified on each ECM analyzed was performed using the tool BiNGO® [36] within Cytoscape® [37], using Benjamini&Hochberg FDR *p* value correction (significance level 0.05).

### 2.4. Cellular *In Vitro* Studies

Human bone marrow-derived mesenchymal cells (hBMMSCs) were provided by the Center for Cell-Based Therapy at USP (CTC-USP). hBMMSCs were kept in alpha minimum essential medium (*α*MEM) supplemented with 10% (*v*/*v*) fetal bovine serum (FBS, ThermoFisher) and 1% (*v*/*v*) antibiotic-antimycotic solution (ABAM, ThermoFisher) (proliferation medium) under standard conditions (37°C, 95% air/5% CO_2_, humidified atmosphere). Cells were used during passage 4, and the culture medium was exchanged every 2 days.

#### 2.4.1. Cellular Adhesion

Cell adhesion experiments were conducted as follows: hBMMSCs were seeded in 24-well plates in a concentration of 3 × 10^4^ cell/well and incubated for 4 h in a proliferation medium supplemented with digested ECMp after the continuous and stepwise method. After that, the medium was collected to remove the nonattached cells, and the metabolic activity of the adhered cells was measured using the Alamar Blue assay [38].

#### 2.4.2. Cellular Proliferation

Proliferation profiles were obtained as described previously [38]: hBMMSCs were seeded in 24-well plates in a concentration of 1 × 10^4^ cell/well and incubated in a proliferation medium supplemented with digested ECMp, after the continuous and stepwise method. The metabolic activity was measured 5, 8, 12, and 15 days after starting the experiment.

#### 2.4.3. Cellular Differentiation

hBMMSCs were seeded in 24-well plates in the concentration of 5 × 10^4^ cell/well and incubated for 48 h in a proliferation medium. After that, an osteogenic medium (proliferation medium with *β*-glycerophosphate (2.5 mM), dexamethasone (1 *μ*M), and ascorbic 2-phosphate (280 *μ*M)) supplemented with digested ECMp, after the continuous and stepwise method, was added.

The osteogenic differentiation of hBMMSCs was evaluated in two experiments, (1) by quantifying the activity of alkaline phosphatase (ALP) and (2) by the mineralization capacity.

After 9, 14, and 21 days, ALP activity was quantified by using the p-Nitrophenyl Phosphate Liquid Substrate System (N7653, Sigma-Aldrich) according to the manufacturer's instructions. The cells were washed twice with PBS and incubated in the dark for 30 min with 0.25 mL of ALP substrate at 37°C, allowing the conversion of pNPP to pNP (yellow) by alkaline phosphatase. The solution absorbance was measured at *λ* = 405 nm in the Infinite 200 Pro NanoQuant (Tecan, Switzerland) microplate reader. This assay was performed in three different concentrations of ECM digestion products: 5 *μ*g/mL, 25 *μ*g/mL, and 250 *μ*g/mL to evaluate the initial concentration at which the cells start to respond to the presence of digested ECMp.

As a late marker, the mineralization was assessed only after 14 and 21 days using Alizarin red staining (ARS). The cells were incubated with an osteogenic medium supplemented with 5 *μ*g/mL of ECMp digested. After 14 and 21 days in culture, cells were washed twice with PBS and incubated for 20 min with fixating solution (formaldehyde in PBS (10% *v*/*v*)), followed by PBS wash and 5 min incubation with staining solution (Alizarin red staining (1% *w*/*v*), ethanol (2% *v*/*v*) in water, pH 4.2). The stained material was dried at room temperature and solubilized with cetylpyridinium chloride solution (10% *w*/*v*) [39]. Calcium ions were quantified by measuring absorbance at 575 nm using an Infinite 200 Pro NanoQuant (Tecan, Switzerland) microplate reader.

For statistical purposes, all results (adhesion, proliferation, and differentiation) were determined in quintuplicate. Normality was evaluated using Shapiro-Wilk's test, and differences between groups were all assessed via Student's *t*-test with significance criteria assuming a 95% confidence level (*p* < 0.05).

## 3. Results

**Figure 1:**
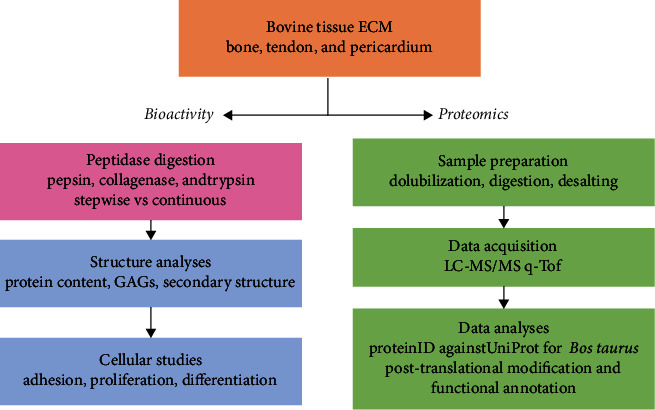
Flowchart of the work rationale. The aim is to compare a stepwise versus continuous digestion (pink), to evaluate the conservation of active sites and binding ligands of proteins of interest. The final products were characterized by their capability to promote/inhibit cell adhesion, proliferation, and differentiation (blue). A proteomic analysis (green) was performed for the different decellularized ECM samples to investigate their original biological potential.

The rationale of the work is presented in Figure 1 and comprises (i) the evaluation of the bioactivity of three commercial decellularized ECMs after being unfolded by stepwise and continuous digestion; the digestion products were quantified regarding total protein content, total GAG content, and the presence of protein's secondary structures, as well as evaluated regarding their influence on cellular adhesion, proliferation, and differentiation, and (ii) accessing the original proteomic content of each decellularized ECM to evaluate its functional potential.

### 3.1. Characterization of Digested ECMs

Through peptides and GAG quantification, it is possible to identify how different proteases, as well as the digestion methods, reshape the initial ECM. Three proteases, trypsin, pepsin, and collagenase, and two digestion methods, continuous and stepwise, were used to test this hypothesis.

**Figure 2:**
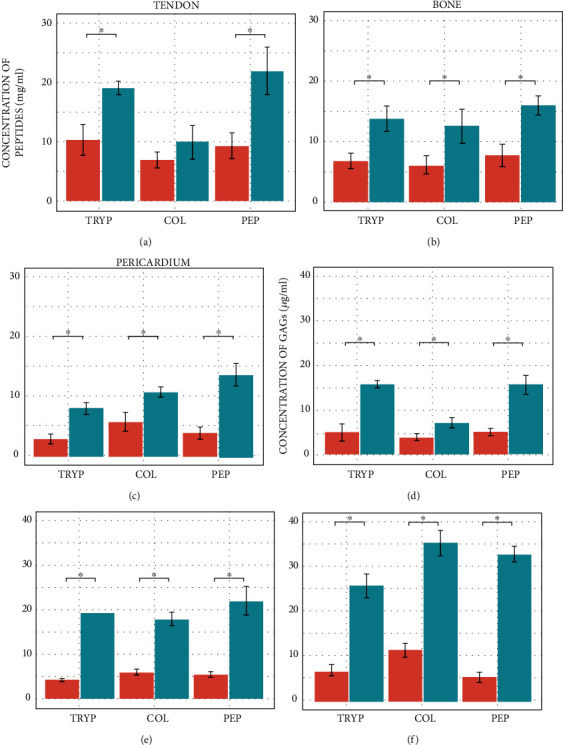
Barplots of peptides (a–c) and GAG (d–f) concentration after ECMt (a, d), ECMb (b, e), and ECMp (c, f) digestion with collagenase (left), pepsin (center), and trypsin (right) using continuous (red) and stepwise (blue) methods. The star (∗) shows the statistical difference between treatments (*p* < 0.05).

Results show which digestion protocol provides more significant conservation of protein and GAG structures responsible for the biological responses. Peptide quantification was performed using the BCA assay [40], which identifies peptide bonds. The higher the concentration in Figures 2(a)–2(c), the lower the protein's degradation. The analysis showed that the stepwise digestion method was statistically superior compared to the continuous process, implying that the stepwise method is a better option to preserve the protein structure (Student's *t*-test *p* value = 0.1345). Also, Figures 2(a)–2(c) as well as Figures 2(d)–2(f) show the impact of different proteases and the ECM sources on the obtained mixture.

The exception was observed for ECMt digested with collagenase, probably due to tendon composition and the *C. histolyticum* collagenase capability to dismantle collagen. Unlike mammalian collagenase, such as matrix metalloproteinases (MMP), which show specificity towards each collagen and tightly regulated cleavage sites, *C. histolyticum* collagenases can digest most collagen types efficiently. It hydrolyzes multiple sites within the triple helix backbone and digests the obtained peptides in oligopeptides, due to its tripeptidyl-carboxypeptidase activity [41]. Thus, as fibrous collagen is the most abundant protein in the tendon, approximately 75% of its dry weight [42], it is reasonable to expect high degradation rates, regardless of the method.

Pepsin seems to be a common protease that leads to a significant release of peptides for all ECMs analyzed. While for ECMt, pepsin and trypsin led to a minor degradation than collagenase, for ECMb, all proteases lead to nearly the same amount of degradation. For ECMp, collagenase and pepsin yielded a less degraded product than trypsin. It is also clear that the resulting protein mixture is, as expected, different from each ECM protease combination.

Collagen is known to be resistant to most protease activity, not only due to its triple-helix structure, which hides the cleavage sites in cryptic pockets [41], but also due to the primary sequence of the *α* chains, with a high content of Gly, Pro, and Hyp [43]. An exception is *C. histolyticum* collagenase that hydrolyzes peptides with the sequence X-Pro-R-Gly-Pro-Y (X, Y, and R could be any amino acid residue) in the R-Gly bond [44–46]. Thus, it is expected that digestion by this collagenase yields more denatured protein fragments in comparison to the other proteases, corroborating the results observed in the BCA assay.

GAGs are linear chains of carbohydrate polymers present on the cell surface and within the ECM, linked to proteins forming proteoglycans. Its ability to interact with a wide range of proteins and cellular modulators (chemokines, cytokines, and growth factors) grants them a strategic role in metabolic pathways such as enzymatic regulation, cell adhesion, growth, migration, differentiation, and coagulation [47]. Thus, its presence in the digestion product was considered an indicator of the potential biological activity of the material. GAG quantification was possible due to the metachromasia phenomenon that occurs when the original blue color of the DMMB changes to pink when it attaches itself to polyanionic substrates [48] and O- and N-sulfated GAG portions.

Once again, the analysis showed that the stepwise digestion method was statistically superior to the continuous method for all types of digestion (Figures 2(d)–2(f)). Also, the analysis shows that pericardium releases more GAGs than bone and tendon ECMs, confirming that these molecules are abundant in cardiac tissue and are imperative to create a loose and hydrated matrix required during essential events in heart development and remodeling [49]. Different from Figures 2(a)–2(c), Figures 2(d)–2(f) show that the protease activity is not a determinant of the total GAG content, in contrast to the ECM source and the digestion method.

**Figure 3:**
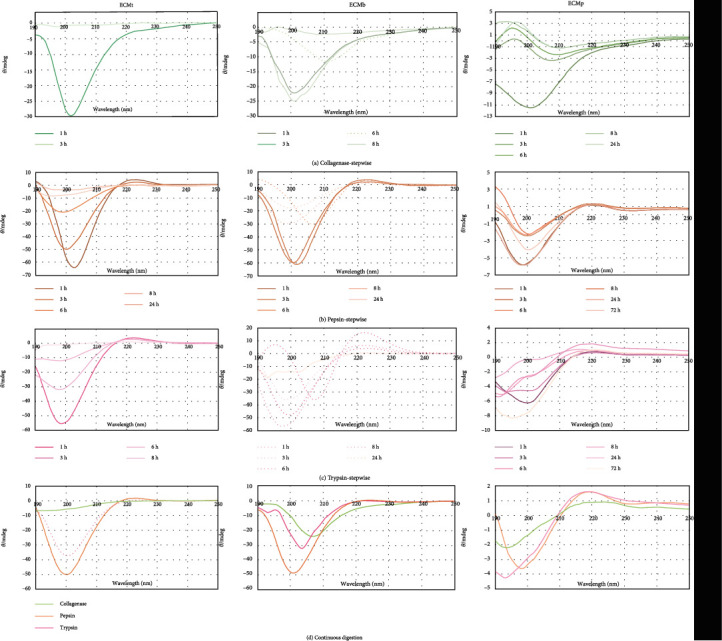
Circular dichroism spectra regarding ECMt, ECMb, and ECMp after continuous and stepwise digestion.

Circular dichroism spectroscopy was used to verify secondary structures in solution aimed at an evaluation of structure preservation. As described in Table 1, as well as in the spectra in Figure 3, stepwise digestion preserves more the secondary structures than continuous digestion as expected. Also, one can observe that for all the ECM sources, in at least one type of digestion (considering enzyme and digestion method), a clockwise triple helix, known as poly-l-proline type II (PPII), was identified. For example, X, Y, and (1) ECMt: continuous digestion with pepsin and stepwise digestion with pepsin and trypsin; (2) ECMo: stepwise digestion with pepsin and trypsin; (3) ECMp: continuous digestion with pepsin, trypsin, and collagenase, as well as stepwise digestion with pepsin and trypsin.

This structure is standard in collagen fibers and is formed by folding three long peptide chains (chains *α*). The *α* chains present a glycine every three residues (-Gly-X-Y-), supporting its tight entanglement (less steric hindrance offered by glycine). X and Y may be any amino acids; however, the X-position is usually a proline, owing to its ring structure which stabilizes the helix, and Y, hydroxyproline [50].

Due to the insertion of hydroxyl groups in proline and lysine (hydroxyproline and hydroxylysine), it is possible to form hydrogen bonds between the *α* chains, stabilizing the triple helix structure, also known as tropocollagen (TC). TC molecules hierarchically bind to each other to form macroscopic resistant fibers observed in bone, tendon, and basement membrane [50]. Thus, the presence of the PPII motif in those ECM samples confirms that, after decellularization, its collagen content was preserved, once fibrous proteins, especially collagen, are abundant in the extracellular environment, aiding in the adhesion of cells and other proteins in the tissue formation [51].

### 3.2. Proteome Analysis of Digested ECMs

The shotgun proteome analysis of the bovine ECMs derived from tendon, bone, and pericardium revealed that the three ECM extracts contain a different proteome profile (Table 2). The proteome analysis revealed the identity of the protein repertoire of the ECM from pericardium (92 proteins identified), ECM from tendon (48 proteins identified), and ECM from bone (38 proteins identified). These results indicate that the functional role of each ECM is associated with its specific protein composition, and it may be synthesized dynamically for the specialized needs of each tissue [51–54]. Therefore, the identification of the proteomic profile of each ECM material contributes to the understanding of the specificities and protein or peptide requirements of each original tissue, where the ECM was extracted. It suggests how the cells adhere to this material and how they interact with each other and with their microenvironment.

Due to the metabolic control that the ECM provides, some proteins need to undergo transcriptional (exon replication, alternative splicing), translational, and posttranslational controls to become functional components of ECM [34, 55]. Thus, in this study, the most frequent posttranslational modifications (PTMs) described in the literature were included in our proteomic analysis, including disulfide bridges (Cys-Cys), deamination (N), hydroxyproline oxidation (M), phosphorylation (P), and sulfation (S) [34].

The list of proteins identified for each ECM and the results of the analyses targeted to the identification of PTMs are reported together with the list of all peptides identified in these analyses (see Supporting Information, Table S1). In total, 182 proteins were identified, from which 123 were identified as having peptides containing PTMs. In the functional annotation of the complete proteome set and the PTM data analysis, the Gene Ontology annotations were retrieved for the two protein datasets. Also, a global Gene Ontology enrichment analysis was performed to identify the biological processes, molecular function, and cellular component GO categories associated with the whole set of proteins identified (Supporting Information). This analysis was performed using the BiNGO© tool from Cytoscape© [56]. GO annotation enrichment analysis was performed for GO categories of biological processes and molecular function terms for the datasets of proteins identified in each ECM. Biological and experimental associated information for specific proteins was analyzed using the Integrity platform (Clarivate Analytics®).

The enrichment analysis of GO terms revealed that, although the three different ECMs showed a very diverse proteome profile, the proteomes of the three ECMs have as most significantly enriched terms “skin morphogenesis” (GO_ID: 43589; corrected *p* value 4.75*E* − 3) and “epidermis development” (GO_ID: 8544; corrected *p* value 3.02*E* − 2), which are both associated to the presence of the collagen alpha-1 (I) chain and collagen alpha-2 (I) chain proteins in the three ECMs analyzed. Of note, our proteomic analysis showed that all three types of ECMs contain collagen alpha-1 (I) chain and collagen alpha-2 (I) chain proteins with posttranslational modifications. Both collagen proteins were detected with deamidation and oxidation sites, but hydroxyproline sites were only identified in the collagen alpha-1 (I) chain, while phosphorylation sites were identified only in the collagen alpha-2 (I) chain. However, nonmodified versions of the collagen alpha-1 (I) chain and collagen alpha-2 (I) samples were detected only for the tendon samples.

Collagen type I is the primary component of a highly organized fiber structure in most connective tissues. It is a heterotrimer comprised of three polypeptide chains, two collagen alpha-1 (I) chain units, and one unit of collagen alpha-2 (I) encoded by the COL1A1 and COL1A2 genes, respectively [57]. The triple helix configuration is through intramolecular hydrogen bonding provided by hydroxyl groups in the hydroxyproline [58, 59]. Besides, the presence of this PTM is necessary to promote integrin binding due to its presence in the binding motif of collagen receptor integrin, as well as structural stabilization [60].

During collagen fibril formation, lysine residues are deaminated by lysyl oxidase (LOX) to form aldehydic residues (*α*-aminoadipic acid-*δ*-semialdehyde) while specific tyrosine residues are oxidized. These highly reactive groups initiate cross-link reactions intra- and intermolecular that increase the biomechanical stiffness of the fibril [61, 62]. Apart from molecular regulation, scaffold stiffness can regulate cell growth, viability, motility, and differentiation, as well as the degree of cell-matrix adhesion [63]. Although the regulatory mechanisms of most of these events are unknown, Vora et al. linked N-terminal deamination with tumor suppression and cell proliferation inhibition [64]. Even though different ECMs require different types and amounts of collagen according to their necessity, as expected and shown in Tables S3–S5, in Supporting Information, all three have collagen alpha-1 (I) chain and collagen alpha-2 (I) chain, due to the basic composition of the collagen fibril.

Moreover, other enriched GO terms of the category biological process showed slightly different lists of significant terms for the different proteome profiles of the ECMs, indicating that ECMs from the different tissues may exert different functional roles on tissue biogenesis and maintenance. The list of the significantly enriched GO terms of the category molecular function for each ECM proteome is shown in Table S1 (Supporting Information). The proteome analysis indicates that pericardium ECM has a more diverse set of proteins, including proteins reported to be essential for the normal collagen fibrillogenesis and development of connective tissue. Examples are the following: mimecan, a protein from the small leucine-rich proteoglycan (SLRP) gene family [65]; prolargin, a proline-arginine-rich end leucine-rich repeat protein (PRELP) that anchors basement membranes to connective tissue [66], proteins related to the early development of heart muscle, such as myomesin 2 [67, 68] and pericentrin [69, 70]; and transcription factors that regulate the expression of cardiac genes encoding structural and regulatory proteins of cardiomyocytes, such as cardiac transcription factor 1 and transcription factor GATA-5 [71]. This indicates that pericardium ECM has a broader range of protein species related to the underlying mechanisms of muscle development.

### 3.3. Biologic Activity

The pericardium was chosen as the sole tissue to be tested, given the protein profile identified through proteomic analysis.

#### 3.3.1. Adhesion and Proliferation

**Figure 4:**
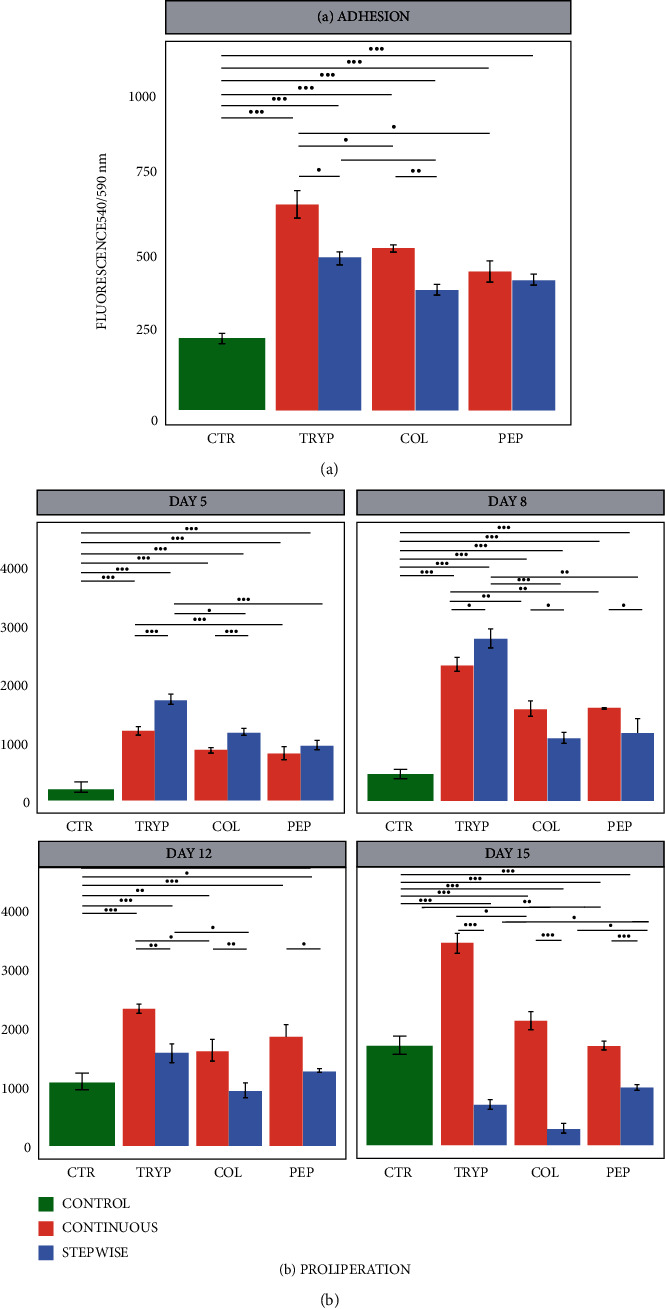
Barplots regarding the (a) adhesion and (b) proliferation processes. Adhesion was accessed after 4 h of experiment and proliferation was measured for 15 days, both using hBMMSCs incubated with ECMp digested with trypsin, collagenase, and pepsin using continuous (red) and stepwise (blue) methods. The star (∗) shows the statistical difference between treatments (*p* < 0.05).

The influence of ECMp digested material upon cell adhesion and proliferation on untreated PS plate was evaluated. It was observed that all treatments provided a significantly higher adhesion than the control, suggesting that there are peptides capable of enhancing this process (Figure 4(a)). It was observed that, by comparing the OD of the enzymes and the digestion methods, the continuous method presents greater adhesion potential for trypsin and collagenase digests. In contrast, no difference for pepsin was observed. In addition, it is possible to note that trypsin was superior to the other enzymes in both methods.

The proteomic analysis (see Supporting Information, Table S2) showed that ECMp has proteins that may regulate cell-matrix adhesion in its composition, such as collagen IV, laminin, *α*-tubulin I, TGFBI (transforming growth factor-*β*-induced protein Ig-h3), von Willebrand factor (vWF), and SRLPs as lumican, prolargin, fibromodulin, and mimecan [72, 73]. Also, the analysis identified chains of collagen *α*1 (I) and collagen *α*2 (I), proteins that have anchoring sites for MSCs (collagen receptor integrin-binding motif) [60], while the digestion process possibly helped to expose these sites [72, 74].

The effect of ECMp was also used to assess the capacity to improve cell proliferation in an environment free of osteogenic inductors (Figure 4(b)). Time points from days 5, 8, 12, and 15 were acquired. Up to day 12, all treatments provided a significantly higher proliferation rate than the control, suggesting that there are peptides inducing cell division, except for the samples treated with collagenase-ECMp digested using the stepwise method.

The cell growth profiles were significantly different for the two digestion methods. The continuous method showed an increase in cell proliferation during all 15 days of the experiment. In contrast, the stepwise method showed rapid growth in the first 8 days and a decrease in the metabolic activity on the last days of analysis. Noteworthy, it was observed that the trypsin digested ECMp showed the highest efficiency in enhancing cell proliferation.

Once again, the proteomic analysis showed (Supporting Information, Table S2) that the ECMp presents proteins that may enhance cell division, like TGIF (transforming growth interacting factor) [75, 76], FYVE [77–79], MAP3K1 (mitogen-activated protein kinase 1) [80], and Par6 [81]. However, proteins known to inhibit cell proliferation were also found, such as NIMA-related kinase 11 (never in mitosis, gene A) [82, 83], and apoptosis-associated tyrosine kinase (AATK) [84]. Proteins that regulate cell migration and ECM remodeling were also found, as ADAMTS1 (a disintegrin and metalloprotease with thrombospondin motif) and MMP14 (matrix metalloproteinase-14) [85], reflecting the complexity of the protein mixture found.

#### 3.3.2. Osteogenic Differentiation

We examined hBMMSC differentiation for 21 days when the cells were cultured in an osteogenic medium supplemented with digested ECMp to determine the protease type and digestion method's effects on the metabolic processes. The main goal of this study is to analyze the role of the digested ECM in the remodeling of bone tissue. Its capacity to differentiate MSCs in preosteoblast was selected as an essential characteristic. This initial stage occurs between days 5 and 15 after the addition of stimuli, and it is characterized, among other things, by the expression of ALP.

**Figure 5:**
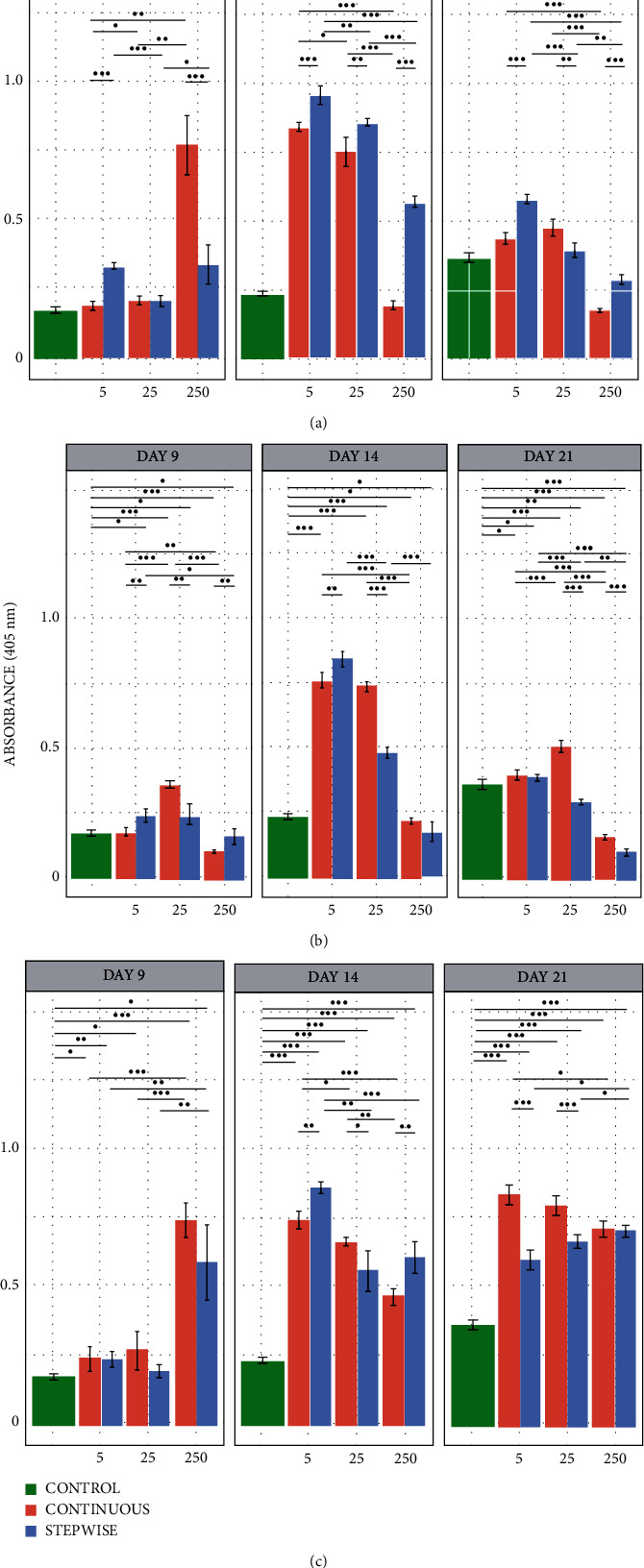
Barplots regarding initial osteoblastic differentiation measured indirectly by ALP activity. The experiment was performed for 21 days, using hBMMSCs incubated with ECMp digested with trypsin (a), collagenase (b), and pepsin (c) after continuous (red) and stepwise (blue) methods. The star (∗) shows the statistical difference between treatments (*p* < 0.05).

Figure 5 shows the absorbance measured at *λ* = 405 nm, characteristic of the conversion of pNPP to pNP, after incubation of the hBMMSCs with the ECMp mixture after digestion with trypsin, collagenase, and pepsin, respectively. When trypsin and collagenase were used as a digestion enzyme, a peak of ALP activity was observed after 14 days of culture, followed by a decrease in its activity as expected [86, 87]. It was observed that the samples treated with 5 *μ*g/mL of ECMp digested with trypsin and collagenase exhibited higher ALP activity than those treated with 25 and 250 *μ*g/mL for both continuous and stepwise methods. Furthermore, it is noted that the stepwise method for trypsin is about 10% more efficient than the continuous method in samples treated with 5 *μ*g/mL (day 14). However, the stepwise treatment is only 5% more efficient for collagenase.

On the other hand, ALP activity did not present a typical expression profile when pepsin was used instead [86, 87]. There was an increase in ALP activity on day 14, remaining constant after that. On day 14, samples treated with 5 *μ*g/mL of digested ECMp showed higher absorbance than the other concentrations for continuous and stepwise methods. On the 21^st^ day, however, the 5 *μ*g/mL and 25 *μ*g/mL samples did not show any difference, and the continuous treatment was superior. In comparison, when using 250 *μ*g/mL of extract, very early ALP expression was observed, only after 9 days of incubation, remaining constant for the subsequent 21 days.

It is possible to note an increase in ALP activity from day 9 to day 14 and the decrease of their levels on day 21 for the digestion methods using trypsin or collagenase, suggesting a complete process of differentiation and maturation of the osteoblasts is operative [86, 87]. For pepsin, on the other hand, there is a progressive increase in the ALP activity, suggesting a delay in osteoblastic maturation.

Another well-established evidence for osteogenesis is the formation of calcium phosphate crystals. During the final differentiation stage, the deposition of hydroxyapatite crystals occurs in the organic matrix of the bone in an organized way and can be quantified using the Alizarin red assay (ARS). The mineralization is regulated by the structure of the collagen fibrils, as well as by other proteins and proteoglycans capable of initial nucleation, such as the case of sialoproteins and dentin [88].

**Figure 6:**
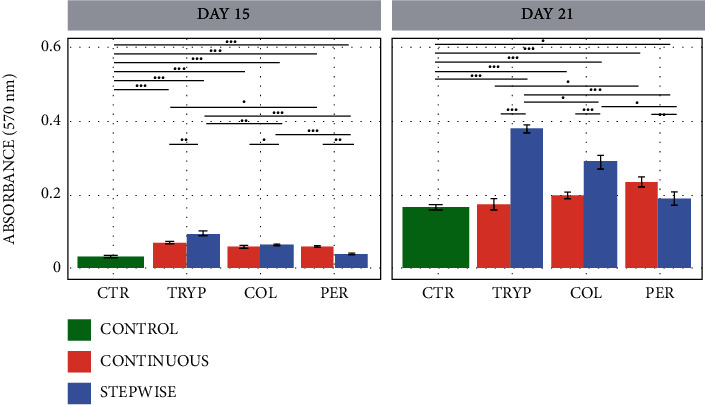
Barplots regarding final osteoblastic differentiation measured by cell mineralization using Alizarin red staining. The experiment was performed for 21 days, using hBMMSCs incubated with ECMp digested with trypsin, collagenase, and pepsin after continuous (red) and stepwise (blue) methods. The star (∗) shows the statistical difference between treatments (*p* < 0.05).

As expected, Figure 6 shows higher mineralization after 21 days of treatment, when the osteoblasts are already mature and expressing nucleating proteins. The results also show that the digestion method used affects the production of active peptides. The continuous method produces a mixture that induces higher mineralization for pepsin, followed by collagenase and trypsin as digestion enzymes. Conversely, the stepwise method produces the most bioactive mixture when trypsin and collagenase are in the digestion step.

Corroborating the ALP activity data, in which cells treated with trypsin and collagenase digests showed a reduction in their activity after 21 days, higher mineralization for the stepwise method was observed, suggesting that these materials promote a more efficient differentiation than the pepsin digest.

## 4. Discussion

The search for materials with effective regenerative abilities focused on tissue repair is still a challenge. Several products that are currently available or under study are not yet able to mimic the intrinsic biochemical morphology of tissues. Specifically for bone repairing and remodeling, current materials present inadequate composition, texturing, 3D architecture of natural bone, and reabsorption hindrances, making it difficult to enable satisfactory bone regeneration [27, 89]. For this reason, new approaches using biological material from animals or *in vitro* cell culture have been considered.

Extracellular matrixes from different tissues can be very distinct regarding their composition and structural arrangement, generating unique materials, from high elasticity to calcified forms. They contain different amounts of various types of collagens, glycoproteins, proteoglycans, and other structuring proteins. More importantly, they also present different types and quantities of growth factors like BMPs, VEGF, FGF, and TNFs and molecules like enzymes and chemokines [1]. This diversity also enables the required 3D structure and porosity appropriate for cell scaffolds, promoting its adhesion, survival, homeostasis, proliferation, repair, morphogenesis, migration, and differentiation. ECM's mechanical stimuli (different rigidity) and the release of soluble factors can trigger intra- and intercellular signaling, regulating the aforementioned processes [1, 38, 89].

However, it has been verified that the application of rigid supports for ECM can do more harm than benefits to the patient, due to tissue damage resulting from the invasive application, leading to its clinical failure. Facing this challenge, attempts have been made to solubilize decellularized ECM and create materials capable of being injected, such as powders and gels [1, 51]. To do so, several proteases have been tested [7] [12],^–^ [14, 16, 29, 30], but only a few of these studies have presented significant conclusions on the most effective protocol to control cell response from the digested product. Additionally, these new materials showed a rapid biodegradation rate and low mechanical strength [7, 12–14].

Here, tissues from three different origins—bone, pericardium, and tendon—were chosen to be evaluated regarding the osteogenic ability of their digestion product. They were subjected to total digestion by two methods—continuous and stepwise—by three different proteases: trypsin, pepsin, and collagenase. The main idea is to optimize the bioinductive capacity of any ECM-derived material, seeking alternative ways to solubilize the proteins therein present, without, however, degrading them to the point of losing their function. In other words, the rationale is that the stepwise digestion would maintain the integrity of the amino acid sequence of protein active sites and binding ligands while freeing them from the main ECM framework.

Our results have shown that stepwise digestion was more efficient regarding peptide bond preservation, amount of GAGs, and secondary structures of proteins in solution. In parallel, bone marrow mesenchymal stem cells incubated with 5 *μ*g/mL of stepwise digested products showed higher differentiation rates compared to continuous digestion. For trypsin and collagenase digests, cells have shown higher ALP activity after 14 days of incubation and higher mineralization after 21 days, suggesting that both of them promoted differentiation from BMMSC to osteoblast, as well as their maturation. Pepsin was the exception, although the stepwise digestion products also promoted greater ALP activity on day 14, while remaining constant until day 21, suggesting that there was no final preosteoblast maturation. In addition, the mineralization rate was lower than observed for other enzymes and the same between digestion methods.

The higher osteoinductive potential presented by the digests may be related to the presence of mimecan (osteoglycine) in ECMp. This proteoglycan is secreted in the ECM by osteoblasts, acting with TGF-*β*1 and 2 in the induction of mineralization, collagen fibrillogenesis, and regulation of bone mass [65, 90]. Studies with MC3T3-E1 cells suggest that osteoglycine can enhance the expression of ALP, Col1, *β*-catenin, and osteocalcin, arresting the expression of Runx2 and osterix in osteoblasts to induce osteoblast mineralization and maturation [91]. However, Runx2 induces differentiation in early stages and also acts negatively on the differentiation of preosteoblasts into mature osteoblasts [92]. In addition, it was observed in the CD experiments that in several samples of digested ECMp, there were triple helix fragments from collagen fibrils, which were also identified in the proteomic analysis. Studies conducted by Salasznyk et al. show that MSCs cultured in the presence of collagen I enter the process of osteogenic differentiation, mediated by integrins and laminins, activating the ERK1/2 signaling pathway leading to the expression of osteopontin and osteocalcin [93, 94].

For cell adhesion tests, continuous treatment showed to be more efficient for trypsin and collagenase, while for trypsin no significant differences between the two digestion methods were observed. For proliferation, nonetheless, the stepwise digestion is more efficient in the first 5 days of experiment, being replaced by continuous treatment after 8 days of incubation and promoting cell detachment from day 15 onwards. Hence, it is possible to affirm that stepwise digestion is able to preserve more active structures than continuous digestion, but this does not necessarily mean that it will be more efficient for all cellular processes involved in tissue regeneration. Differently, the type of protease tends to show a pattern of efficiency among the different experiments, with trypsin being more efficient, followed by collagenase and then pepsin. However, proteomic and gene activation studies of intracellular components are necessary to understand how the material is activating or inhibiting metabolic pathways of BMMSCs, as well as the secreted content, to establish which factors are being released into the medium and how they influence the surrounding microenvironment.

Although the aim here is to create possibilities for the development of alternative materials for bone tissue regeneration, the proteomic analysis showed that these ECMs have the potential to trigger the differentiation of mesenchymal cells into cells other than osteoblasts, such as adipose tissue, cardiac tissue, and possibly epithelial cells, due to a rich diversity of signaling proteins present. Since the matrices were of commercial origin and we did not have access or control over the decellularization process used, it was not possible to assess the degree of degradation to which the material was exposed. Therefore, we strongly believe that a more controlled decellularization process may lead to an even richer set of proteins.

Also, this study raises the possibility of using the described methods to build customized 3D scaffolds. In order to elucidate the mechanism behind antineoplastic effects of trabectedin on undifferentiated pleomorphic sarcoma (UPS) and L-sarcomas, De Vita et al. used three different approaches: patient-derived primary cell culture in 2D and plain 3D collagen hydrogels and *in vivo* experiments (zebrafish model). Their group experienced differences in cell sensitivity for all tested drugs when varying from 2D to 3D culture. They speculate that these results may be due to trabectedin and other drugs, specific microenvironment modulation, including remodeling of ECM [95]. It is possible to suggest from this work that more accurate 3D gels for drug testing could be developed, considering the ECM biochemical composition and the specificities of each tissue.

To conclude, associating these possibilities with the evolution of techniques such as 3D printing, it would be possible to design tissue-specific bioinks that after being printed would exhibit the desired biochemical, rheological, and microstructure properties. In addition, this alternative would enable the creation of personalized structures for patients.

## 5. Conclusion

Two decellularized ECM digestion methods were performed (continuous and stepwise digestion), aiming to optimize the bioinductive ability of the final peptide mixture, searching for alternative ways to solubilize the proteins present in the ECM minimizing the loss of its functionalities. The results here show that stepwise digestion was more efficient in preserving peptide bonds, the total amount of GAGs in solution, and the secondary structures of proteins than continuous digestion. However, this aspect does not necessarily mean that using stepwise digestion will be more efficient to trigger a cellular response as adhesion, proliferation, differentiation, migration, and tissue remodeling. Results have shown that changing the selected protease tends to establish an efficiency pattern.

While we focused on bone remodeling aspects, the proteomic analysis indicates that the material composition has more influence on cellular response than the origin of the tissue from which the ECM was extracted. This is crucial, since ECM goes through decellularization before it is suitable for tissue engineering application, and decellularization might lead to protein degradation and consequently loss of bioinductivity. This explains the reason why ECMp was selected over ECMb for cellular assays. Additionally, proteomics have shown that based on what was identified from ECMp, ECMb, and ECMt, the differentiation of mesenchymal cells may be possible for other cell types as well, such as adipose, cardiac, and possibly epithelial cells, due to the presence of related signaling and growth factor proteins.

Finally, it is clear that despite knowing the initial protein content from the commercial ECMs, to understand the intricated metabolic pathways that its proteins and peptides (after digestion) can trigger, it is imperative to perform proteomic studies of cells that were incubated with ECM digestion products for several days. Its intracellular components, in comparison with the control, would show which biological processes are being upregulated or downregulated. Likewise, it would be of utmost importance to replicate these experiments with the culture medium, to verify which factors are being released in the medium and how they influence their microenvironment.

To conclude, with this work presents new possibilities for tissue-engineering material, with a highly customizable composition, varying its bioinductive composition, by selecting the ECM, the digestive enzyme, and the type of digestion. These discoveries, associated with new technologies, may add different mechanical properties to it, designed according to the application.

## Figures and Tables

**Table 1:** List of secondary structures that were identified during circular dichroism analysis performed after each digestion method, using ECM from tendon (ECMt), bone (ECMb), and pericardium (ECMp) and pepsin, trypsin, and collagenase as proteases.

ECM	Enzyme	Continuous digestion	Stepwise digestion
Tendon	Pepsin	PPII	PPII
	Trypsin	Denatured protein	PPII
	Collagenase	Denatured protein	Denatured protein

Bone	Pepsin	Denatured protein	PPII
	Trypsin	Denatured protein	PPII and *α*-helix
	Collagenase	Denatured protein	Denatured protein

Pericardium	Pepsin	PPII	PPII and *β*-sheet or *α*-helix
	Trypsin	PPII	PPII
	Collagenase	PPII	*β*-Sheet

**(a):** 

Molecular function Gene Ontology enriched categories for ECMt proteome profile				
GO-ID	*p* value	Corr *p* value	Description	Proteins in test set
48407	8.71 × 10^−6^	3.57 × 10^−4^	Platelet-derived growth factor binding	P02465|P02453
5201	7.06 × 10^−5^	1.45 × 10^−3^	Extracellular matrix structural constituent	P02465|P02453
19838	4.81 × 10^−4^	6.57 × 10^−3^	Growth factor binding	P02465|P02453
51059	4.86 × 10^−3^	4.99 × 10^−2^	NF-kappaB binding	Q24JZ4

**(b):** 

Molecular function Gene Ontology enriched categories for ECMb proteome profile				
GO-ID	*p* value	Corr *p* value	Description	Proteins in test set
48407	6.22 × 10^−6^	2.37 × 10^−4^	Platelet-derived growth factor binding	P02465|P02453
5201	5.05 × 10^−5^	6.40 × 10^−4^	Extracellular matrix structural constituent	P02465|P02453
30674	5.05 × 10^−5^	6.40 × 10^−4^	Protein binding, bridging	Q17QL6|P02465
19838	3.45 × 10^−4^	3.27 × 10^−3^	Growth factor binding	P02465|P02453
30280	5.97 × 10^−4^	4.54 × 10^−3^	Structural constituent of epidermis	Q17QL6
5198	1.26 × 10^−3^	8.00 × 10^−3^	Structural molecule activity	Q17QL6|P02465|P02453
5200	3.58 × 10^−3^	1.94 × 10^−2^	Structural constituent of cytoskeleton	Q17QL6
4383	4.17 × 10^−3^	1.98 × 10^−2^	Guanylate cyclase activity	P19687
9975	7.73 × 10^−3^	3.20 × 10^−2^	Cyclase activity	P19687
42802	9.89 × 10^−3^	3.20 × 10^−2^	Identical protein binding	P02465|P02453
16849	1.01 × 10^−2^	3.20 × 10^−2^	Phosphorus-oxygen lyase activity	P19687
46332	1.01 × 10^−2^	3.20 × 10^−2^	SMAD binding	P02465
17124	1.42 × 10^−2^	4.16 × 10^−2^	SH3 domain binding	A6QR40

**(c):** 

Molecular function Gene Ontology enriched categories for ECMp proteome profile				
GO-ID	*p* value	Corr *p* value	Description	Proteins in test set
48407	9.53 × 10^−5^	1.13 × 10^−2^	Platelet-derived growth factor binding	P02465|P02453
5201	7.64 × 10^−4^	3.72 × 10^−2^	Extracellular matrix structural constituent	P02465|P02453
3958	2.19 × 10^−3^	3.72 × 10^−2^	NADPH-hemoprotein reductase activity	Q3SYT8
4478	2.19 × 10^−3^	3.72 × 10^−2^	Methionine adenosyltransferase activity	Q2KJC6
3979	2.19 × 10^−3^	3.72 × 10^−2^	UDP-glucose 6-dehydrogenase activity	P12378
19862	2.19 × 10^−3^	3.72 × 10^−2^	IgA binding	P00978
16603	2.19 × 10^−3^	3.72 × 10^−2^	Glutaminyl-peptide cyclotransferase activity	Q28120

## Data Availability

The mass spectrometry raw data is deposited in the PeptideAtlas® repository and can be accessed by the code (PASS01440).

## Supplementary Materials

Supplementary Materials Supporting Information: an independent file is provided containing the following detailed information: Table S1: mass spectrometry-based protein identification and posttranslational modification data and Gene Ontology annotation for protein subsets identified in the different samples analyzed. Supp S1a: protein identifications by shotgun mass spectrometry. Supp S1b: identifications of proteins with the following posttranslational modifications: Cys-Cys, hydroxyproline, sulfation (Y), deamination (N), phosphorylation (ST), and oxidation (M). Supp T2c: Gene Ontology enriched categories for the whole set of identified proteins. Supp T2d: Gene Ontology enriched categories for the set of proteins with identified posttranslational modifications (PTMs). Supp T2e: set of all peptides identified by shotgun mass spectrometry. Supp T2f: set of peptides identified in proteins with posttranslational modifications (PTMs). Supp T2g: Gene Ontology annotation for the whole set of identified proteins. Supp T2h: Gene Ontology annotation for the set of proteins with posttranslational modifications (PTMs). Supp T2i: GO terms enriched for the set of proteins identified in the ECMt. Supp T2j: GO terms enriched for the set of proteins identified in the ECMb. Supp T2k: GO enriched for the set of proteins identified in the ECMp. Table S2: significance values for cell adhesion and proliferation assays. Significant *p* values for Student's *t*-test (*α* = 0.05): (a) cell adhesion after 4 h of incubation and (b) cell proliferation after 4, 8, 12, and 15 days. These measurements were performed using the Alamar Blue assay (BMMSCs MO-58) after continuous and stepwise digestion with trypsin, collagenase, and pepsin. Table S3: significance values for peptides and glycosaminoglycan quantification. Significant *p* values for Student's *t*-test (*α* = 0.05) in order to compare: (a–c) peptide quantification using BCA assay and (d, e) GAG quantification using DMMB assay, performed under continuous and stepwise digestion processes. Table S4: significance values for *in vitro* osteogenic differentiation analysis via alkaline phosphatase. Significant *p* values for Student's *t*-test (*α* = 0.05) of ALP activity after 9, 14, and 21 days. BMMSCs MO-58 were incubated with the digestion product ECMp with trypsin, collagenase, and pepsin, performed under continuous and stepwise digestion processes. Table S5: significance values for mineralization capacity. Significant *p* values for Student's *t*-test (*α* = 0.05) of mineralization using ARS after 14 and 21 days. BMMSCs MO-58 were incubated with the digestion product ECMp with trypsin, collagenase, and pepsin, performed under continuous and stepwise digestion processes.
