# Supplementary material for: Proteomic Analysis of Decellularized Extracellular Matrix: Achieving a Competent Biomaterial for Osteogenesis
Source: Biomed Res Int. 2022 Oct 11;2022:6884370. doi: 10.1155/2022/6884370 (PMC9578822; doi:10.1155/2022/6884370)
Supplement: Supplementary Materials — Supporting Information: an independent file is provided containing the following detailed information: Table S1: mass spectrometry-based protein identification and posttranslational modification data and Gene Ontology annotation for protein subsets identified in the different samples analyzed. Supp S1a: protein identifications by shotgun mass spectrometry. Supp S1b: identifications of proteins with the following posttranslational modifications: Cys-Cys, hydroxyproline, sulfation (Y), deamination (N), phosphorylation (ST), and oxidation (M). Supp T2c: Gene Ontology enriched categories for the whole set of identified proteins. Supp T2d: Gene Ontology enriched categories for the set of proteins with identified posttranslational modifications (PTMs). Supp T2e: set of all peptides identified by shotgun mass spectrometry. Supp T2f: set of peptides identified in proteins with posttranslational modifications (PTMs). Supp T2g: Gene Ontology annotation for the whole set of identified proteins. Supp T2h: Gene Ontology annotation for the set of proteins with posttranslational modifications (PTMs). Supp T2i: GO terms enriched for the set of proteins identified in the ECMt. Supp T2j: GO terms enriched for the set of proteins identified in the ECMb. Supp T2k: GO enriched for the set of proteins identified in the ECMp. Table S2: significance values for cell adhesion and proliferation assays. Significant p values for Student's t-test (α = 0.05): (a) cell adhesion after 4 h of incubation and (b) cell proliferation after 4, 8, 12, and 15 days. These measurements were performed using the Alamar Blue assay (BMMSCs MO-58) after continuous and stepwise digestion with trypsin, collagenase, and pepsin. Table S3: significance values for peptides and glycosaminoglycan quantification. Significant p values for Student's t-test (α = 0.05) in order to compare: (a–c) peptide quantification using BCA assay and (d, e) GAG quantification using DMMB assay, performed under continuous and stepwise diges [file 6884370.f1.zip › Supp Table S2c. PROTEOMICS DATA.docx]

| **Supp table 2c. Gene Ontology enrichment analysis for total Proteome profile of ECMs using BiNGO tool** | | | | | | | | | | | | | | |
| --- | --- | --- | --- | --- | --- | --- | --- | --- | --- | --- | --- | --- | --- | --- |
|  |  |  |  |  |  |  |  |  |  |  |  |  |  |  |
|  |  |  |  |  |  |  |  |  |  |  |  |  |  |  |
| **Biological Process Gene Ontology Enriched Categories** | | | | | |  |  |  |  |  |  |  |  |  |
| GO-ID | p-value | corr p-value | x | n | X | N | Description | Genes in test set | |  |  |  |  |  |
| 43589 | 1.16E-05 | 4.75E-03 | 2 | 2 | 29 | 8356 | skin morphogenesis | P02465\|P02453 | |  |  |  |  |  |
| 48730 | 1.73E-04 | 3.02E-02 | 2 | 6 | 29 | 8356 | epidermis morphogenesis | P02465\|P02453 | |  |  |  |  |  |
| 9653 | 3.52E-04 | 3.02E-02 | 7 | 402 | 29 | 8356 | anatomical structure morphogenesis | Q9GLE4\|Q9GK68\|P19687\|P12378\|P02465\|Q3MIB9\|P02453 | | | | | |  |
| 8544 | 3.94E-04 | 3.02E-02 | 3 | 42 | 29 | 8356 | epidermis development | Q17QL6\|P02465\|P02453 | | |  |  |  |  |
| 7398 | 4.84E-04 | 3.02E-02 | 3 | 45 | 29 | 8356 | ectoderm development | Q17QL6\|P02465\|P02453 | | |  |  |  |  |
| 32501 | 4.93E-04 | 3.02E-02 | 12 | 1243 | 29 | 8356 | multicellular organismal process | Q9GLE4\|Q17QL6\|Q2KJ97\|Q28107\|Q9GK68\|P41361\|P19687\|P12378\|P02465\|Q3MIB9\|P02453\|Q7YRQ8 | | | | | | |
| 43588 | 6.27E-04 | 3.02E-02 | 2 | 11 | 29 | 8356 | skin development | P02465\|P02453 | |  |  |  |  |  |
| 7596 | 7.00E-04 | 3.02E-02 | 3 | 51 | 29 | 8356 | blood coagulation | Q28107\|P41361\|Q7YRQ8 | | |  |  |  |  |
| 50817 | 7.00E-04 | 3.02E-02 | 3 | 51 | 29 | 8356 | coagulation | Q28107\|P41361\|Q7YRQ8 | | |  |  |  |  |
| 7599 | 7.41E-04 | 3.02E-02 | 3 | 52 | 29 | 8356 | hemostasis | Q28107\|P41361\|Q7YRQ8 | | |  |  |  |  |
| 30199 | 1.03E-03 | 3.67E-02 | 2 | 14 | 29 | 8356 | collagen fibril organization | P02465\|P02453 | |  |  |  |  |  |
| 48856 | 1.08E-03 | 3.67E-02 | 9 | 797 | 29 | 8356 | anatomical structure development | Q9GLE4\|Q17QL6\|Q2KJ97\|Q9GK68\|P19687\|P12378\|P02465\|Q3MIB9\|P02453 | | | | | | |
| 50878 | 1.24E-03 | 3.89E-02 | 3 | 62 | 29 | 8356 | regulation of body fluid levels | Q28107\|P41361\|Q7YRQ8 | | |  |  |  |  |
|  |  |  |  |  |  |  |  |  |  |  |  |  |  |  |
| **Molecular Function Gene Ontology Enriched Categories** | | | | | |  |  |  |  |  |  |  |  |  |
| GO-ID | p-value | corr p-value | x | n | X | N | Description | Genes in test set | |  |  |  |  |  |
| 48407 | 1.91E-04 | 2.68E-02 | 2 | 7 | 31 | 10055 | platelet-derived growth factor binding | P02465\|P02453 | |  |  |  |  |  |
| 5201 | 1.52E-03 | 4.80E-02 | 2 | 19 | 31 | 10055 | extracellular matrix structural constituent | P02465\|P02453 | |  |  |  |  |  |
| 30674 | 1.52E-03 | 4.80E-02 | 2 | 19 | 31 | 10055 | protein binding, bridging | Q17QL6\|P02465 | |  |  |  |  |  |
| 17124 | 2.43E-03 | 4.80E-02 | 2 | 24 | 31 | 10055 | SH3 domain binding | Q27974\|A6QR40 | |  |  |  |  |  |
| 30280 | 3.08E-03 | 4.80E-02 | 1 | 1 | 31 | 10055 | structural constituent of epidermis | Q17QL6 |  |  |  |  |  |  |
| 3958 | 3.08E-03 | 4.80E-02 | 1 | 1 | 31 | 10055 | NADPH-hemoprotein reductase activity | Q3SYT8 |  |  |  |  |  |  |
| 4478 | 3.08E-03 | 4.80E-02 | 1 | 1 | 31 | 10055 | methionine adenosyltransferase activity | Q2KJC6 |  |  |  |  |  |  |
| 3979 | 3.08E-03 | 4.80E-02 | 1 | 1 | 31 | 10055 | UDP-glucose 6-dehydrogenase activity | P12378 |  |  |  |  |  |  |
| 16603 | 3.08E-03 | 4.80E-02 | 1 | 1 | 31 | 10055 | glutaminyl-peptide cyclotransferase activity | Q28120 |  |  |  |  |  |  |
|  |  |  |  |  |  |  |  |  |  |  |  |  |  |  |
| **Cellular Component Gene Ontology Enriched Categories** | | | | | |  |  |  |  |  |  |  |  |  |
| GO-ID | p-value | corr p-value | x | n | X | N | Description | Genes in test set | |  |  |  |  |  |
| 5584 | 1.10E-05 | 1.05E-03 | 2 | 2 | 30 | 8903 | collagen type I | P02465\|P02453 | |  |  |  |  |  |
| 5583 | 1.63E-04 | 7.84E-03 | 2 | 6 | 30 | 8903 | fibrillar collagen | P02465\|P02453 | |  |  |  |  |  |
| 5581 | 1.28E-03 | 3.54E-02 | 2 | 16 | 30 | 8903 | collagen | P02465\|P02453 | |  |  |  |  |  |
| 5576 | 1.48E-03 | 3.54E-02 | 9 | 854 | 30 | 8903 | extracellular region | Q9GLE4\|Q28107\|P30205\|P0C6R3\|Q9GK68\|P41361\|P02465\|P02453\|Q7YRQ8 | | | | | | |
|  |  |  |  |  |  |  |  |  |  |  |  |  |  |  |
