# Supplementary material for: Proteomic Analysis of Decellularized Extracellular Matrix: Achieving a Competent Biomaterial for Osteogenesis
Source: Biomed Res Int. 2022 Oct 11;2022:6884370. doi: 10.1155/2022/6884370 (PMC9578822; doi:10.1155/2022/6884370)
Supplement: Supplementary Materials — Supporting Information: an independent file is provided containing the following detailed information: Table S1: mass spectrometry-based protein identification and posttranslational modification data and Gene Ontology annotation for protein subsets identified in the different samples analyzed. Supp S1a: protein identifications by shotgun mass spectrometry. Supp S1b: identifications of proteins with the following posttranslational modifications: Cys-Cys, hydroxyproline, sulfation (Y), deamination (N), phosphorylation (ST), and oxidation (M). Supp T2c: Gene Ontology enriched categories for the whole set of identified proteins. Supp T2d: Gene Ontology enriched categories for the set of proteins with identified posttranslational modifications (PTMs). Supp T2e: set of all peptides identified by shotgun mass spectrometry. Supp T2f: set of peptides identified in proteins with posttranslational modifications (PTMs). Supp T2g: Gene Ontology annotation for the whole set of identified proteins. Supp T2h: Gene Ontology annotation for the set of proteins with posttranslational modifications (PTMs). Supp T2i: GO terms enriched for the set of proteins identified in the ECMt. Supp T2j: GO terms enriched for the set of proteins identified in the ECMb. Supp T2k: GO enriched for the set of proteins identified in the ECMp. Table S2: significance values for cell adhesion and proliferation assays. Significant p values for Student's t-test (α = 0.05): (a) cell adhesion after 4 h of incubation and (b) cell proliferation after 4, 8, 12, and 15 days. These measurements were performed using the Alamar Blue assay (BMMSCs MO-58) after continuous and stepwise digestion with trypsin, collagenase, and pepsin. Table S3: significance values for peptides and glycosaminoglycan quantification. Significant p values for Student's t-test (α = 0.05) in order to compare: (a–c) peptide quantification using BCA assay and (d, e) GAG quantification using DMMB assay, performed under continuous and stepwise diges [file 6884370.f1.zip › Supp Table S2d. PROTEOMICS DATA.docx]

| **Supp table 2d. Gene Ontology enrichment analysis of proteins with PTMs profile of ECMs using BiNGO tool** | | | | | | | | | | | | | | |
| --- | --- | --- | --- | --- | --- | --- | --- | --- | --- | --- | --- | --- | --- | --- |
|  |  |  |  |  |  |  |  |  |  |  |  |  |  |  |
| **Biological Process Gene Ontology Enriched Categories** | | | | | |  |  |  |  |  |  |  |  |  |
| GO-ID | p-value | corr p-value | x | n | X | N | Description | Genes in test set | |  |  |  |  |  |
| 43589 | 2.86E-07 | 3.52E-05 | 2 | 2 | 5 | 8356 | skin morphogenesis | P02465\|P02453 | |  |  |  |  |  |
| 48730 | 4.29E-06 | 2.64E-04 | 2 | 6 | 5 | 8356 | epidermis morphogenesis | P02465\|P02453 | |  |  |  |  |  |
| 43588 | 1.57E-05 | 6.45E-04 | 2 | 11 | 5 | 8356 | skin development | P02465\|P02453 | |  |  |  |  |  |
| 30199 | 2.60E-05 | 7.99E-04 | 2 | 14 | 5 | 8356 | collagen fibril organization | P02465\|P02453 | |  |  |  |  |  |
| 10810 | 7.21E-05 | 1.77E-03 | 2 | 23 | 5 | 8356 | regulation of cell-substrate adhesion | A7E303\|P02453 | |  |  |  |  |  |
| 9887 | 1.55E-04 | 3.18E-03 | 3 | 212 | 5 | 8356 | organ morphogenesis | A7E303\|P02465\|P02453 | | |  |  |  |  |
| 8544 | 2.44E-04 | 3.84E-03 | 2 | 42 | 5 | 8356 | epidermis development | P02465\|P02453 | |  |  |  |  |  |
| 7398 | 2.81E-04 | 3.84E-03 | 2 | 45 | 5 | 8356 | ectoderm development | P02465\|P02453 | |  |  |  |  |  |
| 30198 | 2.81E-04 | 3.84E-03 | 2 | 45 | 5 | 8356 | extracellular matrix organization | P02465\|P02453 | |  |  |  |  |  |
| 30155 | 4.05E-04 | 4.98E-03 | 2 | 54 | 5 | 8356 | regulation of cell adhesion | A7E303\|P02453 | |  |  |  |  |  |
| 43062 | 5.17E-04 | 5.78E-03 | 2 | 61 | 5 | 8356 | extracellular structure organization | P02465\|P02453 | |  |  |  |  |  |
| 34505 | 5.98E-04 | 6.13E-03 | 1 | 1 | 5 | 8356 | tooth mineralization | P02453 |  |  |  |  |  |  |
| 32502 | 9.45E-04 | 8.94E-03 | 4 | 1006 | 5 | 8356 | developmental process | A7E303\|Q9GKN8\|P02465\|P02453 | | | |  |  |  |
| 9653 | 1.03E-03 | 9.03E-03 | 3 | 402 | 5 | 8356 | anatomical structure morphogenesis | A7E303\|P02465\|P02453 | | |  |  |  |  |
| 60346 | 1.20E-03 | 9.81E-03 | 1 | 2 | 5 | 8356 | bone trabecula formation | P02453 |  |  |  |  |  |  |
| 1501 | 1.53E-03 | 1.00E-02 | 2 | 105 | 5 | 8356 | skeletal system development | P02465\|P02453 | |  |  |  |  |  |
| 1568 | 1.70E-03 | 1.00E-02 | 2 | 111 | 5 | 8356 | blood vessel development | P02465\|P02453 | |  |  |  |  |  |
| 48729 | 1.77E-03 | 1.00E-02 | 2 | 113 | 5 | 8356 | tissue morphogenesis | P02465\|P02453 | |  |  |  |  |  |
| 1944 | 1.77E-03 | 1.00E-02 | 2 | 113 | 5 | 8356 | vasculature development | P02465\|P02453 | |  |  |  |  |  |
| 1957 | 1.79E-03 | 1.00E-02 | 1 | 3 | 5 | 8356 | intramembranous ossification | P02453 |  |  |  |  |  |  |
| 60351 | 1.79E-03 | 1.00E-02 | 1 | 3 | 5 | 8356 | cartilage development involved in endochondral bone morphogenesis | P02453 |  |  |  |  |  |  |
| 32964 | 1.79E-03 | 1.00E-02 | 1 | 3 | 5 | 8356 | collagen biosynthetic process | P02453 |  |  |  |  |  |  |
| 60343 | 2.39E-03 | 1.28E-02 | 1 | 4 | 5 | 8356 | trabecula formation | P02453 |  |  |  |  |  |  |
| 48513 | 3.22E-03 | 1.58E-02 | 3 | 595 | 5 | 8356 | organ development | A7E303\|P02465\|P02453 | | |  |  |  |  |
| 10812 | 3.59E-03 | 1.58E-02 | 1 | 6 | 5 | 8356 | negative regulation of cell-substrate adhesion | P02453 |  |  |  |  |  |  |
| 18298 | 3.59E-03 | 1.58E-02 | 1 | 6 | 5 | 8356 | protein-chromophore linkage | P00978 |  |  |  |  |  |  |
| 60323 | 3.59E-03 | 1.58E-02 | 1 | 6 | 5 | 8356 | head morphogenesis | P02453 |  |  |  |  |  |  |
| 60325 | 3.59E-03 | 1.58E-02 | 1 | 6 | 5 | 8356 | face morphogenesis | P02453 |  |  |  |  |  |  |
| 60322 | 4.18E-03 | 1.66E-02 | 1 | 7 | 5 | 8356 | head development | P02453 |  |  |  |  |  |  |
| 60324 | 4.18E-03 | 1.66E-02 | 1 | 7 | 5 | 8356 | face development | P02453 |  |  |  |  |  |  |
| 10171 | 4.18E-03 | 1.66E-02 | 1 | 7 | 5 | 8356 | body morphogenesis | P02453 |  |  |  |  |  |  |
| 7569 | 4.78E-03 | 1.78E-02 | 1 | 8 | 5 | 8356 | cell aging | Q9GKN8 |  |  |  |  |  |  |
| 1958 | 4.78E-03 | 1.78E-02 | 1 | 8 | 5 | 8356 | endochondral ossification | P02453 |  |  |  |  |  |  |
| 60350 | 5.38E-03 | 1.92E-02 | 1 | 9 | 5 | 8356 | endochondral bone morphogenesis | P02453 |  |  |  |  |  |  |
| 48731 | 5.47E-03 | 1.92E-02 | 3 | 715 | 5 | 8356 | system development | A7E303\|P02465\|P02453 | | |  |  |  |  |
| 32963 | 6.57E-03 | 2.24E-02 | 1 | 11 | 5 | 8356 | collagen metabolic process | P02453 |  |  |  |  |  |  |
| 44259 | 7.16E-03 | 2.32E-02 | 1 | 12 | 5 | 8356 | multicellular organismal macromolecule metabolic process | P02453 |  |  |  |  |  |  |
| 50673 | 7.16E-03 | 2.32E-02 | 1 | 12 | 5 | 8356 | epithelial cell proliferation | A7E303 |  |  |  |  |  |  |
| 48856 | 7.46E-03 | 2.35E-02 | 3 | 797 | 5 | 8356 | anatomical structure development | A7E303\|P02465\|P02453 | | |  |  |  |  |
| 7266 | 8.35E-03 | 2.51E-02 | 1 | 14 | 5 | 8356 | Rho protein signal transduction | P02465 |  |  |  |  |  |  |
| 7162 | 8.35E-03 | 2.51E-02 | 1 | 14 | 5 | 8356 | negative regulation of cell adhesion | P02453 |  |  |  |  |  |  |
| 60349 | 8.95E-03 | 2.56E-02 | 1 | 15 | 5 | 8356 | bone morphogenesis | P02453 |  |  |  |  |  |  |
| 44236 | 8.95E-03 | 2.56E-02 | 1 | 15 | 5 | 8356 | multicellular organismal metabolic process | P02453 |  |  |  |  |  |  |
| 10811 | 9.54E-03 | 2.67E-02 | 1 | 16 | 5 | 8356 | positive regulation of cell-substrate adhesion | A7E303 |  |  |  |  |  |  |
| 9888 | 9.82E-03 | 2.68E-02 | 2 | 271 | 5 | 8356 | tissue development | P02465\|P02453 | |  |  |  |  |  |
| 7179 | 1.07E-02 | 2.69E-02 | 1 | 18 | 5 | 8356 | transforming growth factor beta receptor signaling pathway | P02465 |  |  |  |  |  |  |
| 1649 | 1.07E-02 | 2.69E-02 | 1 | 18 | 5 | 8356 | osteoblast differentiation | P02453 |  |  |  |  |  |  |
| 7568 | 1.07E-02 | 2.69E-02 | 1 | 18 | 5 | 8356 | aging | Q9GKN8 |  |  |  |  |  |  |
| 31214 | 1.07E-02 | 2.69E-02 | 1 | 18 | 5 | 8356 | biomineral formation | P02453 |  |  |  |  |  |  |
| 7275 | 1.13E-02 | 2.78E-02 | 3 | 922 | 5 | 8356 | multicellular organismal development | A7E303\|P02465\|P02453 | | |  |  |  |  |
| 42476 | 1.25E-02 | 3.02E-02 | 1 | 21 | 5 | 8356 | odontogenesis | P02453 |  |  |  |  |  |  |
| 48706 | 1.31E-02 | 3.03E-02 | 1 | 22 | 5 | 8356 | embryonic skeletal system development | P02453 |  |  |  |  |  |  |
| 48593 | 1.31E-02 | 3.03E-02 | 1 | 22 | 5 | 8356 | camera-type eye morphogenesis | A7E303 |  |  |  |  |  |  |
| 3008 | 1.33E-02 | 3.03E-02 | 2 | 317 | 5 | 8356 | system process | P02465\|P02453 | |  |  |  |  |  |
| 7605 | 1.37E-02 | 3.06E-02 | 1 | 23 | 5 | 8356 | sensory perception of sound | P02453 |  |  |  |  |  |  |
| 50954 | 1.49E-02 | 3.21E-02 | 1 | 25 | 5 | 8356 | sensory perception of mechanical stimulus | P02453 |  |  |  |  |  |  |
| 45785 | 1.49E-02 | 3.21E-02 | 1 | 25 | 5 | 8356 | positive regulation of cell adhesion | A7E303 |  |  |  |  |  |  |
| 7265 | 1.78E-02 | 3.78E-02 | 1 | 30 | 5 | 8356 | Ras protein signal transduction | P02465 |  |  |  |  |  |  |
| 48592 | 1.96E-02 | 4.09E-02 | 1 | 33 | 5 | 8356 | eye morphogenesis | A7E303 |  |  |  |  |  |  |
| 51216 | 2.02E-02 | 4.14E-02 | 1 | 34 | 5 | 8356 | cartilage development | P02453 |  |  |  |  |  |  |
| 8217 | 2.20E-02 | 4.43E-02 | 1 | 37 | 5 | 8356 | regulation of blood pressure | P02465 |  |  |  |  |  |  |
| 7178 | 2.49E-02 | 4.94E-02 | 1 | 42 | 5 | 8356 | transmembrane receptor protein serine/threonine kinase signaling pathway | P02465 |  |  |  |  |  |  |
|  |  |  |  |  |  |  |  |  |  |  |  |  |  |  |
| **Molecular Function Gene Ontology Enriched Categories** | | | | | |  |  |  |  |  |  |  |  |  |
| GO-ID | p-value | corr p-value | x | n | X | N | Description | Genes in test set | |  |  |  |  |  |
| 48407 | 6.22E-06 | 2.05E-04 | 2 | 7 | 6 | 10055 | platelet-derived growth factor binding | P02465\|P02453 | |  |  |  |  |  |
| 5201 | 5.05E-05 | 8.34E-04 | 2 | 19 | 6 | 10055 | extracellular matrix structural constituent | P02465\|P02453 | |  |  |  |  |  |
| 19838 | 3.45E-04 | 2.91E-03 | 2 | 49 | 6 | 10055 | growth factor binding | P02465\|P02453 | |  |  |  |  |  |
| 42802 | 3.53E-04 | 2.91E-03 | 3 | 268 | 6 | 10055 | identical protein binding | P00978\|P02465\|P02453 | | |  |  |  |  |
| 19862 | 5.97E-04 | 3.94E-03 | 1 | 1 | 6 | 10055 | IgA binding | P00978 |  |  |  |  |  |  |
| 5515 | 2.30E-03 | 1.27E-02 | 6 | 3655 | 6 | 10055 | protein binding | P19879\|P00978\|Q05443\|Q9GKN8\|P02465\|P02453 | | | | |  |  |
| 19865 | 3.58E-03 | 1.69E-02 | 1 | 6 | 6 | 10055 | immunoglobulin binding | P00978 |  |  |  |  |  |  |
| 5518 | 5.36E-03 | 2.21E-02 | 1 | 9 | 6 | 10055 | collagen binding | Q05443 |  |  |  |  |  |  |
| 46332 | 1.01E-02 | 3.70E-02 | 1 | 17 | 6 | 10055 | SMAD binding | P02465 |  |  |  |  |  |  |
| 30674 | 1.13E-02 | 3.72E-02 | 1 | 19 | 6 | 10055 | protein binding, bridging | P02465 |  |  |  |  |  |  |
|  |  |  |  |  |  |  |  |  |  |  |  |  |  |  |
| **Cellular Component Gene Ontology Enriched Categories** | | | | | |  |  |  |  |  |  |  |  |  |
| GO-ID | p-value | corr p-value | x | n | X | N | Description | Genes in test set | |  |  |  |  |  |
| 5583 | 3.40E-09 | 4.36E-08 | 3 | 6 | 6 | 8903 | fibrillar collagen | Q05443\|P02465\|P02453 | | |  |  |  |  |
| 5578 | 3.79E-09 | 4.36E-08 | 5 | 131 | 6 | 8903 | proteinaceous extracellular matrix | P19879\|Q05443\|Q9GKN8\|P02465\|P02453 | | | | |  |  |
| 31012 | 8.04E-09 | 6.16E-08 | 5 | 152 | 6 | 8903 | extracellular matrix | P19879\|Q05443\|Q9GKN8\|P02465\|P02453 | | | | |  |  |
| 5581 | 9.49E-08 | 5.46E-07 | 3 | 16 | 6 | 8903 | collagen | Q05443\|P02465\|P02453 | | |  |  |  |  |
| 5584 | 3.79E-07 | 1.74E-06 | 2 | 2 | 6 | 8903 | collagen type I | P02465\|P02453 | |  |  |  |  |  |
| 5576 | 7.67E-07 | 2.94E-06 | 6 | 854 | 6 | 8903 | extracellular region | P19879\|P00978\|Q05443\|Q9GKN8\|P02465\|P02453 | | | | |  |  |
| 44421 | 9.46E-07 | 3.11E-06 | 5 | 393 | 6 | 8903 | extracellular region part | P19879\|Q05443\|Q9GKN8\|P02465\|P02453 | | | | |  |  |
| 44420 | 2.55E-06 | 7.34E-06 | 3 | 46 | 6 | 8903 | extracellular matrix part | Q05443\|P02465\|P02453 | | |  |  |  |  |
|  |  |  |  |  |  |  |  |  |  |  |  |  |  |  |
