# Supplementary material for: Proteomic Analysis of Decellularized Extracellular Matrix: Achieving a Competent Biomaterial for Osteogenesis
Source: Biomed Res Int. 2022 Oct 11;2022:6884370. doi: 10.1155/2022/6884370 (PMC9578822; doi:10.1155/2022/6884370)
Supplement: Supplementary Materials — Supporting Information: an independent file is provided containing the following detailed information: Table S1: mass spectrometry-based protein identification and posttranslational modification data and Gene Ontology annotation for protein subsets identified in the different samples analyzed. Supp S1a: protein identifications by shotgun mass spectrometry. Supp S1b: identifications of proteins with the following posttranslational modifications: Cys-Cys, hydroxyproline, sulfation (Y), deamination (N), phosphorylation (ST), and oxidation (M). Supp T2c: Gene Ontology enriched categories for the whole set of identified proteins. Supp T2d: Gene Ontology enriched categories for the set of proteins with identified posttranslational modifications (PTMs). Supp T2e: set of all peptides identified by shotgun mass spectrometry. Supp T2f: set of peptides identified in proteins with posttranslational modifications (PTMs). Supp T2g: Gene Ontology annotation for the whole set of identified proteins. Supp T2h: Gene Ontology annotation for the set of proteins with posttranslational modifications (PTMs). Supp T2i: GO terms enriched for the set of proteins identified in the ECMt. Supp T2j: GO terms enriched for the set of proteins identified in the ECMb. Supp T2k: GO enriched for the set of proteins identified in the ECMp. Table S2: significance values for cell adhesion and proliferation assays. Significant p values for Student's t-test (α = 0.05): (a) cell adhesion after 4 h of incubation and (b) cell proliferation after 4, 8, 12, and 15 days. These measurements were performed using the Alamar Blue assay (BMMSCs MO-58) after continuous and stepwise digestion with trypsin, collagenase, and pepsin. Table S3: significance values for peptides and glycosaminoglycan quantification. Significant p values for Student's t-test (α = 0.05) in order to compare: (a–c) peptide quantification using BCA assay and (d, e) GAG quantification using DMMB assay, performed under continuous and stepwise diges [file 6884370.f1.zip › Supp Table S2g. PROTEOMICS DATA.docx]

| **Supp table 2g. Functional annotation of proteome profile from synthetic ECMs** | | | | | | |
| --- | --- | --- | --- | --- | --- | --- |
| Protein IDs | PrimeiroDeGO_ID | Description | Sequence coverage % | LFQ intensity col | LFQ intensity ECM0 | LFQ intensity ECMp |
| A0A0A0MP88 | GO:0005615 | Uncharacterized protein | 2.3 | 0 | 28727 | 0 |
| A0JNF4 | GO:0046872 | DNA primase large subunit | 1.8 | 0 | 0 | 1192600 |
| A0N0X6 | GO:0005615 | Leucine-rich repeat neuronal protein 1 | 1.3 | 0 | 0 | 95332 |
| A2VDQ0 | GO:0060271 | Intraflagellar transport 81 | 1.3 | 0 | 0 | 214540 |
| A2VDS4 | GO:0044325 | Potassium inwardly-rectifying channel, subfamily J, member 11 | 2.3 | 0 | 7471.7 | 0 |
| A4FUE5 | GO:1903233 | RAB3 GTPase activating protein catalytic subunit 1 | 1.4 | 0 | 0 | 0 |
| A4IFA8 |  |  | 17.5 | 27541 | 0 | 0 |
| A6QLB8 | GO:1902004 | GSK3A protein | 10.1 | 0 | 9912.1 | 0 |
| A6QLE1 | GO:0010608 | Tudor domain-containing protein 7 | 0.8 | 0 | 0 | 400720 |
| A6QLF4 | GO:0045659 | TRIB1 protein | 11 | 15038 | 0 | 0 |
| A6QLN5 | GO:0005768 | PI4K2A protein | 4.2 | 0 | 418620 | 0 |
| A6QNZ5 | GO:0000226 | MGC151537 protein | 1.2 | 0 | 0 | 117110 |
| A6QP16 | GO:0030177 | Ubiquitin thioesterase ZRANB1 |  | 0 | 0 | 126550 |
| A6QP30 |  |  | 1.6 | 0 | 0 | 221090 |
| A6QPM5 | GO:0016021 | ILDR2 protein | 1.4 | 84388 | 0 | 0 |
| A6QR40 | GO:0017124 | Engulfment and cell motility protein 3 | 1.4 | 0 | 11952 | 0 |
| A7MAZ2 | GO:0005484 | STX12 protein | 3.3 | 0 | 19348 | 0 |
| A7YWN4 | GO:0005634 | Calmodulin-lysine N-methyltransferase | 2.8 | 53190 | 0 | 0 |
| E1B6X6 | GO:0016604 | Bromodomain adjacent to zinc finger domain 1B | 0.6 | 0 | 0 | 289800 |
| E1B754 | GO:0008017 | Microtubule associated serine/threonine kinase 2 | 0.5 | 0 | 285770 | 0 |
| E1B7W2 |  |  | 3.2 | 28287 | 0 | 0 |
| E1B847 | GO:0042981 | Mitogen-activated protein kinase kinase kinase 1 | 0.6 | 0 | 0 | 166190 |
| E1B8C2 | GO:0005730 | Nucleolar protein with MIF4G domain 1 | 1.1 | 0 | 0 | 72911 |
| E1B9Q8 | GO:2000291 | Zinc finger protein 609 | 0.6 | 0 | 7116 | 0 |
| E1BAN6 | GO:0033038 | Taste receptor type 2 | 3.1 | 0 | 0 | 681200 |
| E1BB91 | GO:0004867 | Collagen type VI alpha 3 chain | 0.7 | 0 | 0 | 131670 |
| E1BBW2 |  |  | 1.5 | 0 | 0 | 0 |
| E1BCX0 | GO:0000981 | Ovo like zinc finger 3 | 3.9 | 0 | 0 | 455450 |
| E1BD12 |  |  | 0.7 | 0 | 0 | 226990 |
| E1BD25 | GO:0043565 | B-cell CLL/lymphoma 11B | 5.3 | 6121 | 0 | 0 |
| E1BDM8 | GO:0019074 | DEAD-box helicase 6 | 2.9 | 0 | 0 | 0 |
| E1BE33 | GO:0048066 | Zinc finger E-box binding homeobox 2 | 0.7 | 105090 | 0 | 0 |
| E1BEI7 | GO:0019221 | Reticulon 4 receptor like 1 | 2 | 0 | 0 | 352680 |
| E1BEJ7 | GO:0005829 | Host cell factor C2 | 1.6 | 0 | 0 | 0 |
| E1BEK4 | GO:0005524 | RAD54 like 2 | 0.6 | 0 | 0 | 184060 |
| E1BES5 | GO:0005622 | Uncharacterized protein | 1.7 | 19976 | 0 | 0 |
| E1BFB0 | GO:0043231 | Spectrin alpha, non-erythrocytic 1 | 0.4 | 0 | 0 | 146230 |
| E1BG78 | GO:0035145 | R3H domain and coiled-coil containing 1 like | 1.1 | 51506 | 0 | 0 |
| E1BIF6 |  |  | 0.2 | 0 | 0 | 505260 |
| E1BIR1 | GO:0018105 | Claspin | 0.7 | 0 | 165020 | 0 |
| E1BIT2 | GO:0016021 | Mitochondrial elongation factor 1 | 1.9 | 0 | 0 | 399720 |
| E1BJF4 | GO:0006273 | DNA polymerase | 0.7 | 0 | 0 | 118760 |
| E1BJU4 | GO:0044772 | NIMA related kinase 11 | 1.4 | 0 | 0 | 181040 |
| E1BK89 | GO:0000016 | Lactase | 2.1 | 0 | 0 | 48845 |
| E1BKJ5 | GO:0030914 | Transformation/transcription domain associated protein | 0.2 | 115480 | 0 | 0 |
| E1BKN0 | GO:0016458 | Lysine methyltransferase 2B | 0.3 | 21453 | 0 | 0 |
| E1BKY0 | GO:0003676 | Zinc finger protein 197 | 1.3 | 0 | 0 | 0 |
| E1BKZ0 | GO:0007052 | Pericentrin | 0.3 | 0 | 0 | 118760 |
| E1BL01 | GO:0050885 | Junctophilin 4 | 1.4 | 0 | 16754 | 0 |
| E1BL14 |  |  | 2.7 | 0 | 715460 | 0 |
| E1BLN6 |  |  | 2.5 | 0 | 0 | 0 |
| E1BLP0 | GO:0032991 | PML-RARA regulated adaptor molecule 1 | 1.3 | 0 | 0 | 148180 |
| E1BLY2 | GO:0032508 | Uncharacterized protein | 2.4 | 0 | 0 | 188140 |
| E1BLZ0 | GO:0016579 | Ubiquitinyl hydrolase 1 | 1.3 | 0 | 24991 | 0 |
| E1BM42 |  |  | 1.2 | 0 | 0 | 0 |
| E1BMC4 | GO:0002263 | Golgi brefeldin A resistant guanine nucleotide exchange factor 1 | 0.5 | 0 | 0 | 153630 |
| E1BMV0 | GO:0005198 | Claudin | 4 | 0 | 0 | 237430 |
| E1BN90 | GO:0006351 | Zinc finger with KRAB and SCAN domains 2 | 1.4 | 0 | 0 | 0 |
| E1BND0 | GO:0006511 | Ubiquitin specific peptidase 24 | 0.3 | 38133 | 0 | 0 |
| E1BNH3 | GO:0048488 | Stonin 2 | 1 | 0 | 0 | 216170 |
| E1BNR0 | GO:0042158 | Apolipoprotein B | 0.2 | 0 | 0 | 93380 |
| E1BNZ0 |  |  | 0.9 | 0 | 0 | 0 |
| E1BP31 |  |  | 1.1 | 0 | 0 | 381310 |
| E1BPX1 |  |  | 0.2 | 0 | 0 | 106690 |
| F1MBA0 | GO:0000281 | Cytoskeleton-associated protein 2 | 1.4 | 1464400 | 0 | 0 |
| F1MBS3 | GO:0005615 | Transforming growth factor-beta-induced protein ig-h3 | 1.4 | 0 | 0 | 154800 |
| F1MBW3 | GO:0005739 | Acyl-CoA synthetase long chain family member 4 | 1.2 | 0 | 0 | 596780 |
| F1MCM5 |  |  | 1.9 | 0 | 15088 | 0 |
| F1MD77 | GO:0022617 | Laminin subunit gamma 1 | 0.6 | 0 | 0 | 469220 |
| F1MDF3 | GO:0035249 | ALS2, alsin Rho guanine nucleotide exchange factor | 0.5 | 83776 | 0 | 0 |
| F1MDJ4 | GO:0006893 | Coiled-coil domain containing 93 | 1.4 | 0 | 0 | 92277 |
| F1MF78 | GO:1902017 | Spectrin repeat containing nuclear envelope protein 2 | 0.1 | 0 | 0 | 75391 |
| F1MFR9 | GO:1903078 | Rho guanine nucleotide exchange factor 16 | 1.3 | 0 | 30327 | 0 |
| F1MGL2 | GO:0043547 | ArfGAP with GTPase domain, ankyrin repeat and PH domain 1 | 1.1 | 39914 | 0 | 0 |
| F1MHL9 | GO:0015114 | Xenotropic and polytropic retrovirus receptor 1 | 1.3 | 47416 | 0 | 0 |
| F1MHU8 | GO:0032575 | Immunoglobulin mu binding protein 2 | 0.9 | 0 | 0 | 71140 |
| F1MI56 | GO:0042742 | Ankyrin repeat domain 17 | 0.3 | 0 | 0 | 543060 |
| F1MI83 | GO:0006355 | Mitochondrial transcription termination factor 2 | 2.3 | 259740 | 0 | 0 |
| F1MIB7 | GO:0004672 | Apoptosis associated tyrosine kinase | 0.7 | 0 | 0 | 145190 |
| F1MIU7 | GO:0016021 | Solute carrier family 27 member 3 | 1.3 | 0 | 0 | 462220 |
| F1MJ64 |  |  | 3.4 | 0 | 110590 | 0 |
| F1MKV4 |  |  | 0.5 | 0 | 0 | 372060 |
| F1ML17 | GO:0004888 | NFAT activating protein with ITAM motif 1 | 3.6 | 16306 | 0 | 0 |
| F1ML80 | GO:0001158 | SLC2A4 regulator | 14.1 | 30015 | 0 | 0 |
| F1MLY7 | GO:0032407 | ATR serine/threonine kinase | 0.3 | 0 | 30511 | 0 |
| F1MM78 | GO:0016604 | Zinc finger CCHC-type containing 8 | 2 | 0 | 0 | 0 |
| F1MMP9 | GO:0016021 | Guanylate cyclase | 1.7 | 0 | 0 | 0 |
| F1MP61 | GO:0008270 | Uncharacterized protein | 3.1 | 0 | 0 | 0 |
| F1MPF3 | GO:0005509 | Protocadherin Fat 2 precursor | 0.3 | 0 | 308050 | 0 |
| F1MPW8 | GO:0006355 | Zinc finger homeobox 4 | 0.3 | 0 | 0 | 198520 |
| F1MQ30 | GO:0043087 | FYVE, RhoGEF and PH domain containing 6 | 0.6 | 0 | 0 | 105240 |
| F1MQ44 | GO:0005089 | MCF.2 cell line derived transforming sequence-like 2 | 3.8 | 12600 | 0 | 0 |
| F1MQ65 | GO:0051015 | Myosin IE | 0.8 | 0 | 3311.8 | 0 |
| F1MQ84 | GO:0005815 | Phosphodiesterase 4D interacting protein | 0.4 | 0 | 0 | 28268 |
| F1MRA0 | GO:0005198 | Lamin tail domain containing 1 | 10 | 10505 | 0 | 0 |
| F1MRZ5 |  |  | 0.5 | 0 | 0 | 219480 |
| F1MT78 | GO:0016021 | Endothelial cell adhesion molecule | 2.3 | 0 | 308050 | 0 |
| F1MTH3 | GO:0005737 | T-cell lymphoma invasion and metastasis 2 | 0.5 | 0 | 0 | 426460 |
| F1MU52 |  |  | 2.5 | 0 | 0 | 262610 |
| F1MW01 | GO:0004197 | Extra spindle pole bodies like 1, separase | 0.6 | 0 | 0 | 0 |
| F1MWB8 | GO:0017137 | Dmx like 2 | 0.7 | 0 | 0 | 0 |
| F1MWX8 | GO:0002474 | MHC Class I JSP.1 precursor | 3.9 | 0 | 0 | 0 |
| F1MX94 | GO:0042803 | 6-pyruvoyl tetrahydrobiopterin synthase | 6.2 | 0 | 0 | 371840 |
| F1MY85 | GO:0010575 | Complement C5a anaphylatoxin | 0.5 | 17175 | 0 | 0 |
| F1N0C4 | GO:0005737 | Zinc finger MYND-type containing 19 | 4.3 | 0 | 6085700 | 0 |
| F1N0E0 |  |  | 1 | 0 | 22203 | 0 |
| F1N0F7 | GO:0003743 | Eukaryotic translation initiation factor 5 | 2.1 | 0 | 0 | 135250 |
| F1N0P2 | GO:0005730 | DEAD-box helicase 49 | 2.3 | 147610 | 0 | 0 |
| F1N0Z3 | GO:0005634 | ESX homeobox 1 | 3.2 | 0 | 598760 | 0 |
| F1N1C7 | GO:0004185 | AE binding protein 1 | 0.8 | 0 | 36886 | 0 |
| F1N1C9 | GO:0005634 | Bromodomain and WD repeat domain containing 3 | 0.5 | 0 | 0 | 419190 |
| F1N1E2 | GO:0007163 | Par-6 family cell polarity regulator gamma | 2.4 | 0 | 0 | 166190 |
| F1N1H2 | GO:0030425 | Microtubule-associated protein 1S | 0.8 | 315270 | 0 | 0 |
| F1N1R4 | GO:0005829 | DIS3 homolog, exosome endoribonuclease and 3'-5' exoribonuclease | 0.9 | 0 | 0 | 470450 |
| F1N1Z2 | GO:0030307 | Ubiquitin specific peptidase 47 | 0.7 | 0 | 0 | 50210 |
| F1N222 | GO:0000055 | Protein SDA1 homolog | 1.3 | 0 | 3883400 | 0 |
| F1N2M2 | GO:0071347 | ADAM metallopeptidase with thrombospondin type 1 motif 7 | 0.5 | 35051 | 0 | 0 |
| F1N3I3 | GO:0030513 | Ubiquitin conjugating enzyme E2 O | 3.6 | 10046 | 0 | 0 |
| F1N3I6 | GO:0032587 | WW and C2 domain containing 1 | 0.8 | 26462 | 0 | 0 |
| F1N3U6 | GO:0070418 | UV radiation resistance associated | 1.3 | 0 | 0 | 309610 |
| F1N446 | GO:0005509 | Uncharacterized protein | 0.9 | 239480 | 0 | 0 |
| F1N4J2 | GO:0005739 | Sacsin molecular chaperone | 0.2 | 0 | 592130 | 0 |
| F1N5U1 | GO:0046872 | G2/M-phase specific E3 ubiquitin protein ligase | 2 | 0 | 0 | 0 |
| F1N5X8 | GO:0000209 | RING-type E3 ubiquitin transferase | 3.3 | 47229 | 0 | 0 |
| F1N621 | GO:0005783 | Uncharacterized protein | 2.1 | 0 | 31159 | 0 |
| F1N7E8 | GO:0036064 | Non-specific serine/threonine protein kinase | 1.2 | 25227 | 0 | 0 |
| F1N7F6 | GO:0009986 | Signal peptide, CUB domain and EGF like domain containing 2 | 0.9 | 0 | 0 | 153630 |
| F1N7L8 | GO:0036064 | Basal body orientation factor 1 | 1.7 | 0 | 0 | 2687300 |
| F1N7Z6 | GO:0006508 | Pappalysin 1 | 0.6 | 0 | 0 | 119390 |
| G1K177 | GO:0003723 | Cleavage stimulation factor subunit 2 | 6.4 | 0 | 0 | 45204 |
| G3MWL5 |  |  | 3 | 0 | 0 | 0 |
| G3MWW7 | GO:0045727 | Proline rich 16 | 5.3 | 0 | 0 | 352680 |
| G3MXN7 |  |  | 8.9 | 0 | 0 | 0 |
| G3MXQ9 |  |  | 3 | 0 | 56027 | 0 |
| G3MYU4 |  |  | 1.5 | 0 | 0 | 366710 |
| G3N0T2 | GO:0003676 | Uncharacterized protein | 1.2 | 315270 | 0 | 0 |
| G3N182 | GO:0005737 | Protein phosphatase 1 regulatory subunit 3G | 13.4 | 19388 | 0 | 0 |
| G3N1M8 |  |  | 2.8 | 0 | 0 | 380320 |
| G3N2P1 | GO:0046872 | Zinc finger protein 114 | 2.2 | 483740 | 0 | 0 |
| G3N369 | GO:0003676 | Uncharacterized protein | 1.8 | 0 | 0 | 0 |
| G3N381 |  |  | 1.5 | 0 | 0 | 76023 |
| G3X6U0 |  |  | 1.7 | 0 | 0 | 349740 |
| G5E5L9 | GO:0030036 | IQ motif and Sec7 domain 3 | 0.8 | 0 | 0 | 107310 |
| G5E6N4 | GO:0019706 | Palmitoyltransferase | 2.4 | 0 | 0 | 366830 |
| O02754 | GO:0030324 | CCAAT/enhancer-binding protein alpha | 13.6 | 12519 | 0 | 0 |
| P02453 | GO:0060346 | Collagen alpha-1(I) chain | 1.5 | 12514000 | 0 | 0 |
| P02465 | GO:0070208 | Collagen alpha-2(I) chain | 1.6 | 41561 | 0 | 0 |
| P0C6R3 | GO:0052689 | Carboxylesterase 4A | 1.6 | 69940 | 0 | 0 |
| P12378 | GO:0005829 | UDP-glucose 6-dehydrogenase | 1.8 | 0 | 0 | 108590 |
| P19687 | GO:0008074 | Guanylate cyclase soluble subunit alpha-1 | 1.3 | 0 | 23120 | 0 |
| P30205 | GO:0005044 | Antigen WC1.1 | 0.6 | 0 | 0 | 62441 |
| P41361 | GO:2000266 | Antithrombin-III | 3 | 0 | 0 | 0 |
| Q08E01 | GO:0005911 | Adducin 3 | 2.1 | 0 | 0 | 0 |
| Q0VC20 | GO:0051289 | Retinoic acid receptor RXR-gamma | 1.9 | 0 | 0 | 191100 |
| Q10569 | GO:0006379 | Cleavage and polyadenylation specificity factor subunit 1 | 0.6 | 0 | 0 | 150320 |
| Q148L9 | GO:0016021 | ST6 (Alpha-N-acetyl-neuraminyl-2,3-beta-galactosyl-1, 3)-N-acetylgalactosaminide alpha-2,6-sialyltransferase 2 | 2.6 | 0 | 0 | 235250 |
| Q17QL6 | GO:0030280 | LOR protein | 13.6 | 0 | 9578.3 | 0 |
| Q17QN3 | GO:0005737 | Small nuclear ribonucleoprotein-associated protein N | 20.4 | 30950 | 0 | 0 |
| Q24JZ4 | GO:0031663 | Metadherin | 1.5 | 20883 | 0 | 0 |
| Q27974 | GO:0030276 | Putative tyrosine-protein phosphatase auxilin | 1 | 0 | 0 | 161290 |
| Q28107 | GO:0005507 | Coagulation factor V | 0.6 | 0 | 0 | 0 |
| Q28120 | GO:0005576 | Glutaminyl-peptide cyclotransferase | 2.5 | 0 | 0 | 359690 |
| Q28141 | GO:0048146 | ATP-dependent RNA helicase A | 0.7 | 84388 | 0 | 0 |
| Q29S21 | GO:0005198 | Keratin, type II cytoskeletal 7 | 2.6 | 0 | 0 | 0 |
| Q2KIL9 | GO:0055114 | Estradiol 17-beta-dehydrogenase 12-like | 2.8 | 28054 | 0 | 0 |
| Q2KJ55 | GO:0007015 | Rho GTPase activating protein 25 | 1.4 | 0 | 70861 | 0 |
| Q2KJ97 | GO:0005829 | Neurochondrin | 1.2 | 0 | 0 | 195200 |
| Q2KJC6 | GO:0009087 | S-adenosylmethionine synthase isoform type-1 | 2.3 | 0 | 0 | 303980 |
| Q3MIB9 | GO:0000122 | TGFB induced factor homeobox 1 | 3.6 | 0 | 0 | 547920 |
| Q3SYT8 | GO:0003958 | NADPH--cytochrome P450 reductase | 1.3 | 0 | 0 | 258600 |
| Q3SZ00 | GO:0004300 | HADHA protein | 1.8 | 0 | 0 | 0 |
| Q3SZJ5 | GO:0006366 | Transcription factor GATA-5 | 2.2 | 0 | 0 | 226990 |
| Q3T028 |  |  | 3.3 | 0 | 0 | 218440 |
| Q3T075 | GO:0019869 | Syntaxin-8 | 3.8 | 56442 | 0 | 0 |
| Q3ZCJ7 | GO:0007017 | Tubulin alpha-1C chain | 3.3 | 0 | 0 | 350740 |
| Q56K13 | GO:0000398 | Splicing factor 3B subunit 5 | 10.5 | 0 | 35556 | 0 |
| Q5MD62 | GO:0006954 | C-C chemokine receptor type 7 | 2.4 | 0 | 0 | 13017 |
| Q70IB5 |  |  | 3.5 | 0 | 49127 | 0 |
| Q7YRQ8 | GO:0007596 | Tissue factor pathway inhibitor 2 | 3.8 | 0 | 0 | 81212 |
| Q9GK68 | GO:0010469 | Growth/differentiation factor 9 | 2 | 66153 | 0 | 0 |
| Q9GLE4 | GO:0042470 | Matrix metalloproteinase-14 | 1.5 | 0 | 0 | 282180 |
