# Supplementary material for: Proteomic Analysis of Decellularized Extracellular Matrix: Achieving a Competent Biomaterial for Osteogenesis
Source: Biomed Res Int. 2022 Oct 11;2022:6884370. doi: 10.1155/2022/6884370 (PMC9578822; doi:10.1155/2022/6884370)
Supplement: Supplementary Materials — Supporting Information: an independent file is provided containing the following detailed information: Table S1: mass spectrometry-based protein identification and posttranslational modification data and Gene Ontology annotation for protein subsets identified in the different samples analyzed. Supp S1a: protein identifications by shotgun mass spectrometry. Supp S1b: identifications of proteins with the following posttranslational modifications: Cys-Cys, hydroxyproline, sulfation (Y), deamination (N), phosphorylation (ST), and oxidation (M). Supp T2c: Gene Ontology enriched categories for the whole set of identified proteins. Supp T2d: Gene Ontology enriched categories for the set of proteins with identified posttranslational modifications (PTMs). Supp T2e: set of all peptides identified by shotgun mass spectrometry. Supp T2f: set of peptides identified in proteins with posttranslational modifications (PTMs). Supp T2g: Gene Ontology annotation for the whole set of identified proteins. Supp T2h: Gene Ontology annotation for the set of proteins with posttranslational modifications (PTMs). Supp T2i: GO terms enriched for the set of proteins identified in the ECMt. Supp T2j: GO terms enriched for the set of proteins identified in the ECMb. Supp T2k: GO enriched for the set of proteins identified in the ECMp. Table S2: significance values for cell adhesion and proliferation assays. Significant p values for Student's t-test (α = 0.05): (a) cell adhesion after 4 h of incubation and (b) cell proliferation after 4, 8, 12, and 15 days. These measurements were performed using the Alamar Blue assay (BMMSCs MO-58) after continuous and stepwise digestion with trypsin, collagenase, and pepsin. Table S3: significance values for peptides and glycosaminoglycan quantification. Significant p values for Student's t-test (α = 0.05) in order to compare: (a–c) peptide quantification using BCA assay and (d, e) GAG quantification using DMMB assay, performed under continuous and stepwise diges [file 6884370.f1.zip › Supp Table S2i. PROTEOMICS DATA.docx]

| **Supp table 2i. Enriched Gene Ontology term categories of proteins identified in the ECM col** | | | | | | | | | | | | | |
| --- | --- | --- | --- | --- | --- | --- | --- | --- | --- | --- | --- | --- | --- |
|  |  |  |  |  |  |  |  |  |  |  |  |  |  |
| **Biological Process Gene Ontology Enriched Categories** | | | | | |  |  |  |  |  |  |  |  |
| GO-ID | p-value | corr p-value | x | n | X | N | Description | Genes in test set | |  |  |  |  |
| 43589 | 4.30E-07 | 8.29E-05 | 2 | 2 | 6 | 8356 | skin morphogenesis | P02465\|P02453 | |  |  |  |  |
| 48730 | 6.44E-06 | 6.21E-04 | 2 | 6 | 6 | 8356 | epidermis morphogenesis | P02465\|P02453 | |  |  |  |  |
| 43588 | 2.36E-05 | 1.52E-03 | 2 | 11 | 6 | 8356 | skin development | P02465\|P02453 | |  |  |  |  |
| 30199 | 3.90E-05 | 1.88E-03 | 2 | 14 | 6 | 8356 | collagen fibril organization | P02465\|P02453 | |  |  |  |  |
| 8544 | 3.65E-04 | 1.16E-02 | 2 | 42 | 6 | 8356 | epidermis development | P02465\|P02453 | |  |  |  |  |
| 7398 | 4.20E-04 | 1.16E-02 | 2 | 45 | 6 | 8356 | ectoderm development | P02465\|P02453 | |  |  |  |  |
| 30198 | 4.20E-04 | 1.16E-02 | 2 | 45 | 6 | 8356 | extracellular matrix organization | P02465\|P02453 | |  |  |  |  |
| 1555 | 7.18E-04 | 1.35E-02 | 1 | 1 | 6 | 8356 | oocyte growth | Q9GK68 |  |  |  |  |  |
| 34505 | 7.18E-04 | 1.35E-02 | 1 | 1 | 6 | 8356 | tooth mineralization | P02453 |  |  |  |  |  |
| 48601 | 7.18E-04 | 1.35E-02 | 1 | 1 | 6 | 8356 | oocyte morphogenesis | Q9GK68 |  |  |  |  |  |
| 43062 | 7.72E-04 | 1.35E-02 | 2 | 61 | 6 | 8356 | extracellular structure organization | P02465\|P02453 | |  |  |  |  |
| 9994 | 1.44E-03 | 1.73E-02 | 1 | 2 | 6 | 8356 | oocyte differentiation | Q9GK68 |  |  |  |  |  |
| 70934 | 1.44E-03 | 1.73E-02 | 1 | 2 | 6 | 8356 | CRD-mediated mRNA stabilization | Q28141 |  |  |  |  |  |
| 60346 | 1.44E-03 | 1.73E-02 | 1 | 2 | 6 | 8356 | bone trabecula formation | P02453 |  |  |  |  |  |
| 48588 | 1.44E-03 | 1.73E-02 | 1 | 2 | 6 | 8356 | developmental cell growth | Q9GK68 |  |  |  |  |  |
| 48599 | 1.44E-03 | 1.73E-02 | 1 | 2 | 6 | 8356 | oocyte development | Q9GK68 |  |  |  |  |  |
| 9653 | 1.98E-03 | 2.08E-02 | 3 | 402 | 6 | 8356 | anatomical structure morphogenesis | Q9GK68\|P02465\|P02453 | | |  |  |  |
| 32964 | 2.15E-03 | 2.08E-02 | 1 | 3 | 6 | 8356 | collagen biosynthetic process | P02453 |  |  |  |  |  |
| 1957 | 2.15E-03 | 2.08E-02 | 1 | 3 | 6 | 8356 | intramembranous ossification | P02453 |  |  |  |  |  |
| 60351 | 2.15E-03 | 2.08E-02 | 1 | 3 | 6 | 8356 | cartilage development involved in endochondral bone morphogenesis | P02453 |  |  |  |  |  |
| 1501 | 2.27E-03 | 2.09E-02 | 2 | 105 | 6 | 8356 | skeletal system development | P02465\|P02453 | |  |  |  |  |
| 1568 | 2.53E-03 | 2.11E-02 | 2 | 111 | 6 | 8356 | blood vessel development | P02465\|P02453 | |  |  |  |  |
| 48729 | 2.62E-03 | 2.11E-02 | 2 | 113 | 6 | 8356 | tissue morphogenesis | P02465\|P02453 | |  |  |  |  |
| 1944 | 2.62E-03 | 2.11E-02 | 2 | 113 | 6 | 8356 | vasculature development | P02465\|P02453 | |  |  |  |  |
| 60343 | 2.87E-03 | 2.22E-02 | 1 | 4 | 6 | 8356 | trabecula formation | P02453 |  |  |  |  |  |
| 10812 | 4.30E-03 | 2.68E-02 | 1 | 6 | 6 | 8356 | negative regulation of cell-substrate adhesion | P02453 |  |  |  |  |  |
| 48255 | 4.30E-03 | 2.68E-02 | 1 | 6 | 6 | 8356 | mRNA stabilization | Q28141 |  |  |  |  |  |
| 48477 | 4.30E-03 | 2.68E-02 | 1 | 6 | 6 | 8356 | oogenesis | Q9GK68 |  |  |  |  |  |
| 60323 | 4.30E-03 | 2.68E-02 | 1 | 6 | 6 | 8356 | head morphogenesis | P02453 |  |  |  |  |  |
| 60325 | 4.30E-03 | 2.68E-02 | 1 | 6 | 6 | 8356 | face morphogenesis | P02453 |  |  |  |  |  |
| 43489 | 4.30E-03 | 2.68E-02 | 1 | 6 | 6 | 8356 | RNA stabilization | Q28141 |  |  |  |  |  |
| 60322 | 5.02E-03 | 2.85E-02 | 1 | 7 | 6 | 8356 | head development | P02453 |  |  |  |  |  |
| 60324 | 5.02E-03 | 2.85E-02 | 1 | 7 | 6 | 8356 | face development | P02453 |  |  |  |  |  |
| 10171 | 5.02E-03 | 2.85E-02 | 1 | 7 | 6 | 8356 | body morphogenesis | P02453 |  |  |  |  |  |
| 48523 | 5.54E-03 | 2.91E-02 | 3 | 575 | 6 | 8356 | negative regulation of cellular process | Q24JZ4\|Q9GK68\|P02453 | | |  |  |  |
| 7292 | 5.73E-03 | 2.91E-02 | 1 | 8 | 6 | 8356 | female gamete generation | Q9GK68 |  |  |  |  |  |
| 1958 | 5.73E-03 | 2.91E-02 | 1 | 8 | 6 | 8356 | endochondral ossification | P02453 |  |  |  |  |  |
| 43488 | 5.73E-03 | 2.91E-02 | 1 | 8 | 6 | 8356 | regulation of mRNA stability | Q28141 |  |  |  |  |  |
| 60350 | 6.45E-03 | 3.11E-02 | 1 | 9 | 6 | 8356 | endochondral bone morphogenesis | P02453 |  |  |  |  |  |
| 43487 | 6.45E-03 | 3.11E-02 | 1 | 9 | 6 | 8356 | regulation of RNA stability | Q28141 |  |  |  |  |  |
| 16049 | 7.87E-03 | 3.53E-02 | 1 | 11 | 6 | 8356 | cell growth | Q9GK68 |  |  |  |  |  |
| 32963 | 7.87E-03 | 3.53E-02 | 1 | 11 | 6 | 8356 | collagen metabolic process | P02453 |  |  |  |  |  |
| 34605 | 7.87E-03 | 3.53E-02 | 1 | 11 | 6 | 8356 | cellular response to heat | Q28141 |  |  |  |  |  |
| 48519 | 8.36E-03 | 3.67E-02 | 3 | 665 | 6 | 8356 | negative regulation of biological process | Q24JZ4\|Q9GK68\|P02453 | | |  |  |  |
| 44259 | 8.59E-03 | 3.68E-02 | 1 | 12 | 6 | 8356 | multicellular organismal macromolecule metabolic process | P02453 |  |  |  |  |  |
| 50794 | 8.83E-03 | 3.69E-02 | 5 | 2396 | 6 | 8356 | regulation of cellular process | Q24JZ4\|Q9GK68\|Q28141\|P02465\|P02453 | | | |  |  |
| 9887 | 8.98E-03 | 3.69E-02 | 2 | 212 | 6 | 8356 | organ morphogenesis | P02465\|P02453 | |  |  |  |  |
| 7266 | 1.00E-02 | 3.94E-02 | 1 | 14 | 6 | 8356 | Rho protein signal transduction | P02465 |  |  |  |  |  |
| 7162 | 1.00E-02 | 3.94E-02 | 1 | 14 | 6 | 8356 | negative regulation of cell adhesion | P02453 |  |  |  |  |  |
| 44236 | 1.07E-02 | 4.06E-02 | 1 | 15 | 6 | 8356 | multicellular organismal metabolic process | P02453 |  |  |  |  |  |
| 60349 | 1.07E-02 | 4.06E-02 | 1 | 15 | 6 | 8356 | bone morphogenesis | P02453 |  |  |  |  |  |
| 50789 | 1.25E-02 | 4.51E-02 | 5 | 2580 | 6 | 8356 | regulation of biological process | Q24JZ4\|Q9GK68\|Q28141\|P02465\|P02453 | | | |  |  |
| 7179 | 1.29E-02 | 4.51E-02 | 1 | 18 | 6 | 8356 | transforming growth factor beta receptor signaling pathway | P02465 |  |  |  |  |  |
| 1649 | 1.29E-02 | 4.51E-02 | 1 | 18 | 6 | 8356 | osteoblast differentiation | P02453 |  |  |  |  |  |
| 31214 | 1.29E-02 | 4.51E-02 | 1 | 18 | 6 | 8356 | biomineral formation | P02453 |  |  |  |  |  |
| 48856 | 1.39E-02 | 4.78E-02 | 3 | 797 | 6 | 8356 | anatomical structure development | Q9GK68\|P02465\|P02453 | | |  |  |  |
| 9888 | 1.44E-02 | 4.88E-02 | 2 | 271 | 6 | 8356 | tissue development | P02465\|P02453 | |  |  |  |  |
| 42476 | 1.50E-02 | 4.99E-02 | 1 | 21 | 6 | 8356 | odontogenesis | P02453 |  |  |  |  |  |
|  |  |  |  |  |  |  |  |  |  |  |  |  |  |
| **Molecular Function Gene Ontology Enriched Categories** | | | | | |  |  |  |  |  |  |  |  |
| GO-ID | p-value | corr p-value | x | n | X | N | Description | Genes in test set | |  |  |  |  |
| 48407 | 8.71E-06 | 3.57E-04 | 2 | 7 | 7 | 10055 | platelet-derived growth factor binding | P02465\|P02453 | |  |  |  |  |
| 5201 | 7.06E-05 | 1.45E-03 | 2 | 19 | 7 | 10055 | extracellular matrix structural constituent | P02465\|P02453 | |  |  |  |  |
| 19838 | 4.81E-04 | 6.57E-03 | 2 | 49 | 7 | 10055 | growth factor binding | P02465\|P02453 | |  |  |  |  |
| 51059 | 4.86E-03 | 4.99E-02 | 1 | 7 | 7 | 10055 | NF-kappaB binding | Q24JZ4 |  |  |  |  |  |
