# Supplementary material for: Proteomic Analysis of Decellularized Extracellular Matrix: Achieving a Competent Biomaterial for Osteogenesis
Source: Biomed Res Int. 2022 Oct 11;2022:6884370. doi: 10.1155/2022/6884370 (PMC9578822; doi:10.1155/2022/6884370)
Supplement: Supplementary Materials — Supporting Information: an independent file is provided containing the following detailed information: Table S1: mass spectrometry-based protein identification and posttranslational modification data and Gene Ontology annotation for protein subsets identified in the different samples analyzed. Supp S1a: protein identifications by shotgun mass spectrometry. Supp S1b: identifications of proteins with the following posttranslational modifications: Cys-Cys, hydroxyproline, sulfation (Y), deamination (N), phosphorylation (ST), and oxidation (M). Supp T2c: Gene Ontology enriched categories for the whole set of identified proteins. Supp T2d: Gene Ontology enriched categories for the set of proteins with identified posttranslational modifications (PTMs). Supp T2e: set of all peptides identified by shotgun mass spectrometry. Supp T2f: set of peptides identified in proteins with posttranslational modifications (PTMs). Supp T2g: Gene Ontology annotation for the whole set of identified proteins. Supp T2h: Gene Ontology annotation for the set of proteins with posttranslational modifications (PTMs). Supp T2i: GO terms enriched for the set of proteins identified in the ECMt. Supp T2j: GO terms enriched for the set of proteins identified in the ECMb. Supp T2k: GO enriched for the set of proteins identified in the ECMp. Table S2: significance values for cell adhesion and proliferation assays. Significant p values for Student's t-test (α = 0.05): (a) cell adhesion after 4 h of incubation and (b) cell proliferation after 4, 8, 12, and 15 days. These measurements were performed using the Alamar Blue assay (BMMSCs MO-58) after continuous and stepwise digestion with trypsin, collagenase, and pepsin. Table S3: significance values for peptides and glycosaminoglycan quantification. Significant p values for Student's t-test (α = 0.05) in order to compare: (a–c) peptide quantification using BCA assay and (d, e) GAG quantification using DMMB assay, performed under continuous and stepwise diges [file 6884370.f1.zip › supporting material BMRI-c (1).docx]

Proteomic Analysis of Decellularized Extracellular Matrix: Achieving a Competent Biomaterial for Osteogenesis

Gabriela M. Monteiro-Lobato^*^, Flavia V. Winck*^**^*, Pedro S. T. Russo^***^, Luiz H. Catalani^*^

^*^ Departamento de Química Fundamental, Instituto de Química, Universidade de São Paulo, São Paulo, Brazil

^**^ Departamento de Bioquímica, Instituto de Química, Universidade de São Paulo, São Paulo, Brazil

^***^Departamento de Análises Clínicas e Toxicológicas, Faculdade de Ciências Farmacêuticas, Universidade de São Paulo, São Paulo, Brazil

**Supporting Material**

**Table S1. Mass Spectrometry-based protein identification and post-translational modification data and Gene Ontology annotation for protein subsets identified in the different samples analyzed. Excel file printouts (at the end of this file)**

Table S1a- Protein identifications by shotgun mass spectrometry
Table S1b- Identifications of proteins with the following post-translational modifications: -(Cys-Cys Hydroxyproline, Sulfation (Y), Deamination (N), Phosphorylation (ST), Oxidation (M)

Table S1c- Gene Ontology enriched categories for the whole set of identified proteins

Table S1d- Gene Ontology enriched categories for the set of proteins with identified post-translational modifications (PTMs)

Table S1e- Set of all peptides identified by shotgun mass spectrometry

Table S1f- Set of peptides identified in proteins with post-translational modifications (PTMs)
Table S1g- Gene Ontology annotation for the whole set of identified proteins

Table S1h- Gene Ontology annotation for the set of proteins with post-translational modifications (PTMs)

Table S1i- GO terms enriched for the set of proteins identified in the ECMt

Table S1j- GO terms enriched for the set of proteins identified in the ECMb

Table S1k- GO enriched for the set of proteins identified in the ECMp

**Table S2.  Significance values for cell adhesion and proliferation assays.** Significant p-values for Student ́s t-test (α = 0.05) – (a) Cell adhesion after 4h of incubation and (b) Cell proliferation after 4, 8, 12 and 15 days. These measurements were performed using Alamar Blue assay (BMMSCs MO-58) after continuous and stepwise digestion with Trypsin, Collagenase and Pepsin.

| **Sample 1** | **Sample 2** | **p-value** |
| --- | --- | --- |
| **Adhesion - 4h** | | |
| Trypsin - continuous | Control | 4.9×10^-6^ |
| Trypsin - stepwise | Control | 6.28×10^-8^ |
| Collagenase - stepwise | Control | 3.12×10^-7^ |
| Collagenase - continuous | Control | 1.1×10^-9^ |
| Pepsin - continuous | Control | 2.71x10^-5^ |
| Pepsin - stepwise | Control | 7.82x10^-8^ |
| Trypsin - continuous | Trypsin - stepwise | 1.41×10^-2^ |
| Collagenase - continuous | Collagenase - stepwise | 8.07×10^-3^ |
| Trypsin - continuous | Collagenase - continuous | 4.98×10^-2^ |
| Trypsin - continuous | Pepsin - continuous | 3.29×10^-2^ |
| Trypsin - stepwise | Collagenase - stepwise | 4.42×10^-2^ |
| **Proliferation - Day 4** | | |
| Trypsin - continuous | Control | 7.48×10^-8^ |
| Trypsin - stepwise | Control | 3.09×10^-9^ |
| Collagenase - stepwise | Control | 9.08×10^-8^ |
| Collagenase - continuous | Control | 3.47×10^-6^ |
| Pepsin - continuous | Control | 1.48x10^-5^ |
| Pepsin - stepwise | Control | 6.33x10^-7^ |
| Trypsin - continuous | Trypsin - stepwise | 2.15×10^-3^ |
| Collagenase - continuous | Collagenase - stepwise | 8.05×10^-5^ |
| Trypsin - continuous | Pepsin - continuous | 7.82×10^-4^ |
| Collagenase - stepwise | Trypsin - stepwise | 1.88×10^-2^ |
| Pepsin - stepwise | Trypsin - stepwise | 1.88×10^-4^ |
| **Proliferation - Day 8** | | |
| Trypsin - continuous | Control | 9.84×10^-9^ |
| Trypsin - stepwise | Control | 1.03×10^-7^ |
| Collagenase - stepwise | Control | 4.68×10^-6^ |
| Collagenase - continuous | Control | 1.56×10^-6^ |
| Pepsin - continuous | Control | 7.55x10^-6^ |
| Pepsin - stepwise | Control | 1.75x10^-3^ |
| Trypsin - continuous | Trypsin - stepwise | 1.68×10^-2^ |
| Collagenase - continuous | Collagenase - stepwise | 1.55×10^-2^ |
| Pepsin - continuous | Pepsin - stepwise | 4.66×10^-2^ |
| Trypsin - continuous | Collagenase - continuous | 2.76×10^-3^ |
| Trypsin - continuous | Pepsin - continuous | 3.44×10^-3^ |
| Trypsin - stepwise | Pepsin - stepwise | 6.89×10^-3^ |
| Collagenase - stepwise | Trypsin - stepwise | 1.18×10^-4^ |
| **Proliferation - Day 12** | | |
| Trypsin - continuous | Control | 1.26×10^-6^ |
| Trypsin - stepwise | Control | 9.99×10^-4^ |
| Collagenase - continuous | Control | 1.08×10^-3^ |
| Pepsin - continuous | Control | 1.36x10^-4^ |
| Pepsin - stepwise | Control | 3.41x10^-2^ |
| Trypsin - continuous | Trypsin - stepwise | 3.89×10^-3^ |
| Collagenase - continuous | Collagenase - stepwise | 8.07×10^-3^ |
| Pepsin - continuous | Pepsin - stepwise | 1.08×10^-2^ |
| Trypsin - continuous | Collagenase - continuous | 1.57×10^-2^ |
| Collagenase - stepwise | Trypsin - stepwise | 2.28×10^-2^ |
| **Proliferation - Day 15** | | |
| Trypsin - continuous | Control | 1.31×10^-7^ |
| Trypsin - stepwise | Control | 6.18×10^-6^ |
| Collagenase - continuous | Control | 2.06×10^-3^ |
| Collagenase - stepwise | Control | 1.09×10^-6^ |
| Pepsin - stepwise | Control | 2.14x10^-4^ |
| Trypsin - continuous | Trypsin - stepwise | 5.33×10^-5^ |
| Collagenase - continuous | Collagenase - stepwise | 1.72×10^-4^ |
| Pepsin - continuous | Pepsin - stepwise | 3.09×10^-4^ |
| Trypsin - continuous | Collagenase - continuous | 1.21×10^-2^ |
| Trypsin - continuous | Pepsin - continuous | 1.04×10^-3^ |
| Trypsin - stepwise | Pepsin - stepwise | 1.08×10^-2^ |
| Collagenase - stepwise | Pepsin - stepwise | 4.53×10^-3^ |

**Table S3. Significance values for peptides and glycosaminoglycans quantification.** Significant p-values for Student ́s t-test (α = 0.05) in order to compare – (a-c) Peptide quantification using BCA assay and (d-e) GAGs quantification using DMMB assay, performed under continuous and stepwise digestion processes.

| **Peptides (a-c)** | | |
| --- | --- | --- |
| **ECM** | **Enzyme** | **p-value** |
| ECMt | Trypsin | 5.26×10^-3^ |
| ECMt | Pepsin | 4.76×10^-3^ |
| ECMb | Trypsin | 2.91×10^-3^ |
| ECMb | Pepsin | 4.19×10^-4^ |
| ECMb | Collagenase | 2.05×10^-2^ |
| ECMp | Trypsin | 3.07×10^-4^ |
| ECMp | Pepsin | 4.48×10^-4^ |
| ECMp | Collagenase | 4.87×10^-3^ |
| **GAGs (d-e)** | | |
| ECMt | Trypsin | 5.32×10^-4^ |
| ECMt | Pepsin | 1.64×10^-3^ |
| ECMt | Collagenase | 3.77×10^-3^ |
| ECMb | Trypsin | 1.19×10^-3^ |
| ECMb | Pepsin | 3.09×10^-3^ |
| ECMb | Collagenase | 6.62×10^-5^ |
| ECMp | Trypsin | 1.56×10^-4^ |
| ECMp | Pepsin | 1.88×10^-6^ |
| ECMp | Collagenase | 5.72×10^-5^ |

**Table S4. Significance values for *in vitro* osteogenic differentiation analysis via alkaline phosphatase.** Significant p-values for Student ́s t-test (α = 0.05) of ALP activity after 9, 14 and 21 days. BMMSCs MO-58 were incubated with the digestion product ECMp with Trypsin, Collagenase and Pepsin, performed under continuous and stepwise digestion processes.

| **Sample 1** | **Sample 2** | **p-value** |
| --- | --- | --- |
| **Day 9** | | |
| Trypsin continuous - 25 μg/mL | Control | 3.69×10^-3^ |
| Trypsin continuous - 250 μg/mL | Control | 2.13×10^-4^ |
| Trypsin stepwise - 5 μg/mL | Control | 1.79×10^-8^ |
| Trypsin stepwise - 25 μg/mL | Control | 1.01×10^-2^ |
| Trypsin stepwise - 250 μg/mL | Control | 6.83×10^-3^ |
| Collagenase continuous - 25 μg/mL | Control | 3.45×10^-8^ |
| Collagenase continuous - 250 μg/mL | Control | 7.67×10^-6^ |
| Collagenase stepwise - 5 μg/mL | Control | 2.13×10^-3^ |
| Collagenase stepwise - 25 μg/mL | Control | 1.34×10^-2^ |
| Pepsin continuous - 5 μg/mL | Control | 2.96×10^-2^ |
| Pepsin continuous - 25 μg/mL | Control | 3.61×10^-2^ |
| Pepsin continuous - 250 μg/mL | Control | 2.86×10^-6^ |
| Pepsin stepwise - 5 μg/mL | Control | 6.63×10^-3^ |
| Pepsin stepwise - 250 μg/mL | Control | 2.29x10^-3^ |
| Trypsin continuous - 5 μg/mL | Trypsin continuous - 25 μg/mL | 2.81×10^-2^ |
| Trypsin continuous - 5 μg/mL | Trypsin continuous - 250 μg/mL | 1.08×10^-3^ |
| Trypsin continuous - 25 μg/mL | Trypsin continuous - 250 μg/mL | 1.14×10^-3^ |
| Trypsin stepwise - 5 μg/mL | Trypsin stepwise - 25 μg/mL | 7.99×10^-4^ |
| Trypsin stepwise - 25 μg/mL | Trypsin stepwise - 250 μg/mL | 3.24×10^-2^ |
| Trypsin continuous - 5 μg/mL | Trypsin stepwise - 5 μg/mL | 2.39×10^-7^ |
| Trypsin continuous - 250 μg/mL | Trypsin stepwise - 250 μg/mL | 1.27×10^-4^ |
| Collagenase continuous - 5 μg/mL | Collagenase continuous - 25 μg/mL | 1.26×10^-4^ |
| Collagenase continuous - 5 μg/mL | Collagenase continuous - 250 μg/mL | 1.54×10^-3^ |
| Collagenase continuous - 25 μg/mL | Collagenase continuous - 250 μg/mL | 1.21×10^-5^ |
| Collagenase stepwise - 5 μg/mL | Collagenase stepwise - 250 μg/mL | 3.95×10^-2^ |
| Collagenase continuous - 5 μg/mL | Collagenase stepwise - 5 μg/mL | 1.95×10^-3^ |
| Collagenase continuous - 25 μg/mL | Collagenase stepwise - 25 μg/mL | 1.71×10^-3^ |
| Collagenase continuous - 250 μg/mL | Collagenase stepwise - 250 μg/mL | 9.74×10^-3^ |
| Pepsin continuous - 5 μg/mL | Pepsin continuous - 250 μg/mL | 9.14×10^-6^ |
| Pepsin continuous - 25 μg/mL | Pepsin continuous - 250 μg/mL | 2.28×10^-5^ |
| Pepsin stepwise - 5 μg/mL | Pepsin stepwise - 250 μg/mL | 6.04×10^-3^ |
| Pepsin stepwise - 25 μg/mL | Pepsin stepwise - 250 μg/mL | 6.44×10^-3^ |
| **Day 14** | | |
| Trypsin continuous - 5 μg/mL | Control | 9.49×10^-11^ |
| Trypsin continuous - 25 μg/mL | Control | 1.71×10^-5^ |
| Trypsin continuous - 250 μg/mL | Control | 1.06×10^-3^ |
| Trypsin stepwise - 5 μg/mL | Control | 1.29×10^-7^ |
| Trypsin stepwise - 25 μg/mL | Control | 5.75×10^-14^ |
| Trypsin stepwise - 250 μg/mL | Control | 2.05×10^-7^ |
| Collagenase continuous - 5 μg/mL | Control | 2.81×10^-7^ |
| Collagenase continuous - 25 μg/mL | Control | 9.25×10^-9^ |
| Collagenase continuous - 250 μg/mL | Control | 2.11×10^-2^ |
| Collagenase stepwise - 5 μg/mL | Control | 1.69×10^-7^ |
| Collagenase stepwise - 25 μg/mL | Control | 2.02×10^-6^ |
| **Day 21** | | |
| Trypsin continuous - 5 μg/mL | Control | 4.39×10^-4^ |
| Trypsin continuous - 25 μg/mL | Control | 2.68×10^-4^ |
| Trypsin continuous - 250 μg/mL | Control | 4.44×10^-6^ |
| Trypsin stepwise - 5 μg/mL | Control | 3.69×10^-8^ |
| Trypsin stepwise - 250 μg/mL | Control | 8.12×10^-5^ |
| Collagenase continuous - 5 μg/mL | Control | 1.33x10^-2^ |
| Collagenase continuous - 25 μg/mL | Control | 1.19×10^-5^ |
| Collagenase continuous - 250 μg/mL | Control | 1.61×10^-6^ |
| Collagenase stepwise - 5 μg/mL | Control | 2.32x10^-2^ |
| Collagenase stepwise - 25 μg/mL | Control | 1.54×10^-4^ |
| Collagenase stepwise - 250 μg/mL | Control | 9.22×10^-9^ |
| Pepsin continuous - 5 μg/mL | Control | 3.93x10^-7^ |
| Pepsin continuous - 25 μg/mL | Control | 3.15×10^-7^ |
| Pepsin continuous - 250 μg/mL | Control | 5.28×10^-7^ |
| Pepsin stepwise - 5 μg/mL | Control | 1.36x10^-5^ |
| Pepsin stepwise - 25 μg/mL | Control | 1.85×10^-7^ |
| Pepsin stepwise - 250 μg/mL | Control | 2.09×10^-9^ |
| Trypsin continuous - 25 μg/mL | Trypsin continuous - 250 μg/mL | 5.11×10^-5^ |

**Table S5. Significance values for mineralization capacity.** Significant p-values for Student ́s t-test (α = 0.05) of mineralization using ARS after 14 and 21 days. BMMSCs MO-58 were incubated with the digestion product ECMp with Trypsin, Collagenase and Pepsin, performed under continuous and stepwise digestion processes.

| **Sample 1** | **Sample 2** | **p-valor** |
| --- | --- | --- |
| **Day 14** | | |
| Trypsin - continuous | Control | 1.22×10^-9^ |
| Trypsin - stepwise | Control | 2.15×10^-7^ |
| Collagenase - continuous | Control | 6.96×10^-6^ |
| Collagenase - stepwise | Control | 3.75×10^-9^ |
| Pepsin - continuous | Control | 3.91x10^-8^ |
| Pepsin - stepwise | Control | 4.71x10^-4^ |
| Trypsin - continuous | Pepsin - continuous | 1.85×10^-2^ |
| Trypsin - stepwise | Pepsin - stepwise | 7.19×10^-4^ |
| Trypsin - stepwise | Collagenase - stepwise | 4.79×10^-3^ |
| Collagenase - stepwise | Pepsin - stepwise | 6.16×10^-5^ |
| Trypsin - continuous | Trypsin - stepwise | 2.45×10^-3^ |
| Collagenase - continuous | Collagenase - stepwise | 3.91×10^-2^ |
| Pepsin - continuous | Pepsin - stepwise | 2.89×10^-3^ |
| **Day 21** | | |
| Pepsin - continuous | Control | 7.29x10^-5^ |
| Collagenase - continuous | Control | 2.97×10^-4^ |
| Trypsin - stepwise | Control | 1.41×10^-9^ |
| Pepsin - stepwise | Control | 4.13x10^-2^ |
| Collagenase - stepwise | Control | 1.88×10^-5^ |
| Trypsin - continuous | Pepsin - continuous | 4.83×10^-2^ |
| Trypsin - stepwise | Pepsin - stepwise | 1.55×10^-4^ |
| Trypsin - stepwise | Collagenase - stepwise | 3.1×10^-2^ |
| Collagenase - stepwise | Pepsin - stepwise | 4.12×10^-2^ |
| Trypsin - continuous | Trypsin - stepwise | 3.4×10^-8^ |
| Collagenase - continuous | Collagenase - stepwise | 4.98×10^-5^ |
| Pepsin - continuous | Pepsin - stepwise | 3.16×10^-3^ |
